# Scalable Photoactive NO_2_‐Sensing Framework for Plant Health Monitoring

**DOI:** 10.1002/advs.202518368

**Published:** 2025-11-06

**Authors:** Yun‐Haeng Cho, Kootak Hong, Jung Hwan Seo, Jae Han Chung, Jinho Lee, Sang‐Hyeon Nam, Sunwoo Lee, Jeong‐O Lee, Changui Ahn, Hyojung Kim, Jae Hyun Han, Gyu‐Li Kim, Seong‐Jun Ro, Jun Yeon Hwang, Hyeongyu Gim, Zion Park, Chil‐Hyoung Lee, Dong‐Su Kim, Kwangjae Lee, Young‐Seok Shim, Jun Min Suh, Donghwi Cho

**Affiliations:** ^1^ School of Energy Materials and Chemical Engineering Korea University of Technology and Education Cheonan 31253 Republic of Korea; ^2^ Department of Materials Science and Engineering Chonnam National University Gwangju 61186 Republic of Korea; ^3^ Department of Mechanical Engineering Hongik University 94 Wausan‐ro, Mapo‐gu Seoul 121‐791 Republic of Korea; ^4^ Department of Materials Science and Engineering Korea Advanced Institute of Science and Technology Daejeon 34141 Republic of Korea; ^5^ Department of Material Science and Engineering Massachusetts Institute of Technology Cambridge MA 02139 USA; ^6^ Thin Film Materials Research Center Korea Research Institute of Chemical Technology Daejeon 34114 Republic of Korea; ^7^ Engineering Ceramic Center Korea Institute of Ceramic Engineering and Technology Icheon 17303 Republic of Korea; ^8^ Department of Semiconductor Systems Engineering Sejong University Seoul 05006 Republic of Korea; ^9^ Corning Research & Development Corporation, Corning Asan 31454 Republic of Korea; ^10^ Department of AI Mobility Engineering Sangmyung University Cheonan 31066 Republic of Korea; ^11^ Institute of Advanced Composite Materials Korea Institute of Science and Technology (KIST) Jeonbuk 55324 Republic of Korea; ^12^ National Center for Nano Process & Equipments Energy & NanoTechnology Group Korea Institute of Industrial Technology (KITECH) Gwangju 61012 Republic of Korea; ^13^ Department of Materials Science and Engineering Research Institute of Advanced Materials Seoul National University Seoul 08826 Republic of Korea; ^14^ School of Transdisciplinary Innovations Seoul National University Seoul 08826 Republic of Korea; ^15^ Advanced Materials and Chemical Engineering University of Science and Technology Daejeon 34113 Republic of Korea

**Keywords:** 3D nanoarchitectures, NO_2_ sensors, photoactivation, plant monitoring, room temperature, TiO_2_, wireless microcontrollers

## Abstract

Conventional sensing platforms for plant health monitoring are often limited by high operating temperatures, rigid substrates, and poor compatibility with ambient, power‐constrained, or biologically sensitive environments. These limitations hinder their integration into emerging platforms such as smart agriculture and plant‐interfaced electronics, where mechanical flexibility, energy efficiency, and low thermal budgets are essential. This paper reports a scalable, thermally passive NO_2_ sensor based on light‐activated 3D TiO_2_ nanoarchitectures. Fabricated via sequential glancing angle deposition, the highly ordered porous nanoarchitectures exhibit tunable broadband light scattering and defect‐mediated sub‐bandgap activation under ambient light. Integrated with a wireless microcontroller and mobile application, the sensor enables autonomous NO_2_ monitoring in real‐world conditions. Field deployment on *Mentha suaveolens* plants demonstrates real‐time tracking of gas‐induced physiological stress, establishing practical ecological relevance. This platform overcomes the key limitations of conventional sensors, offering a structurally tunable, spectrally adaptive, and fabrication‐scalable solution for light‐powered, bio‐integrated environmental monitoring.

## Graphical abstract

A highly sensitive and selective NO_2_ gas sensor operates at room temperature using only ambient light, eliminating the need for external heating. Based on a scalable, 3D TiO_2_ nanoarchitecture, the device is integrated into a wireless platform and deployed on a plant. It successfully demonstrates real‐time monitoring of gas‐induced physiological stress, highlighting its potential for smart agriculture and bio‐integrated electronics.

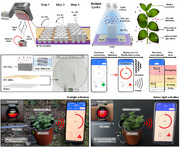

## 1 Introduction

The integration of nanotechnology‐enabled sensors with living plant systems has opened up new avenues for real‐time environmental and physiological monitoring, thereby bridging the gap between biological interfaces and high‐resolution analytical tools.^[^ ^1^ ^]^ Recent advances in optical^[^ ^2^, ^3^, ^4^ ^]^ and electrochemical^[^ ^5^ ^]^ sensing technologies have enabled in situ tracking of phytohormones (e.g., auxin, abscisic acid),^[^ ^6^, ^7^ ^]^ reactive oxygen species (e.g., H_2_O_2_),^[^ ^8^, ^9^, ^10^ ^]^ and nutrient elements (e.g., Ca^2+^, K^+^, Al^3+^),^[^ ^11^, ^12^ ^]^ offering new insights into plant–environment interactions. However, detecting airborne chemical stressors remains technically challenging due to their low concentrations, transient nature, and the limitations of conventional sensors under biological or low‐power conditions.^[^ ^13^, ^14^, ^15^, ^16^, ^17^ ^]^

Nitrogen dioxide (NO_2_), a major byproduct of combustion engines, industrial activity, and fertilizer use, can induce severe oxidative stress in plants even at sub‐ppm levels, reducing photosynthetic efficiency, disrupting respiratory metabolism, and impairing overall plant health.^[^ ^18^, ^19^, ^20^ ^]^ Its impact is particularly significant in urban agricultural settings, where crops are cultivated near pollution sources. Despite its ecological relevance, real‐time, non‐invasive monitoring of NO_2_ in plant environments remains a major unmet need. Although several studies have explored airborne pollutant sensing in plants,^[^ ^5^, ^21^, ^22^ ^]^ conventional platforms often fall short due to their reliance on rigid substrates, high‐temperature operation, or power‐intensive components.^[^ ^23^, ^24^, ^25^, ^26^, ^27^, ^28^, ^29^ ^]^ Moreover, their performance tends to degrade in humid environments, which is common in agricultural settings, leading to poor selectivity and unreliable signals under real‐world conditions.

In this work, we present a light‐activated, thermally passive NO_2_ sensor based on a wafer‐scale 3D TiO_2_ nanoarchitecture, which facilitates scalable fabrication, broadband photoactivation, and compatibility with soft, biological surfaces. By employing a sequential glancing angle deposition (GLAD) technique to construct porous, vertically aligned TiO_2_, we created a 3D photonic structure that combines broadband light scattering, enhanced surface accessibility, and defect‐assisted visible‐light absorption. These features enable efficient photoactivation under ambient light conditions, eliminating the need for dedicated light sources or thermal excitation.^[^ ^30^, ^31^, ^32^ ^]^ Leveraging this ambient‐light functionality, we conducted field trials on living plants (*Mentha suaveolens*), demonstrating autonomous detection of NO_2_‐induced physiological stress. These results establish a direct link between nanomaterial‐based gas sensing and real‐time feedback in agricultural plant systems.

## 2 Results

### 2.1 Scalable 3D TiO_2_ Nanoarchitectures for Thermally Passive Light‐Activated Gas Sensing

**Figure 1:**
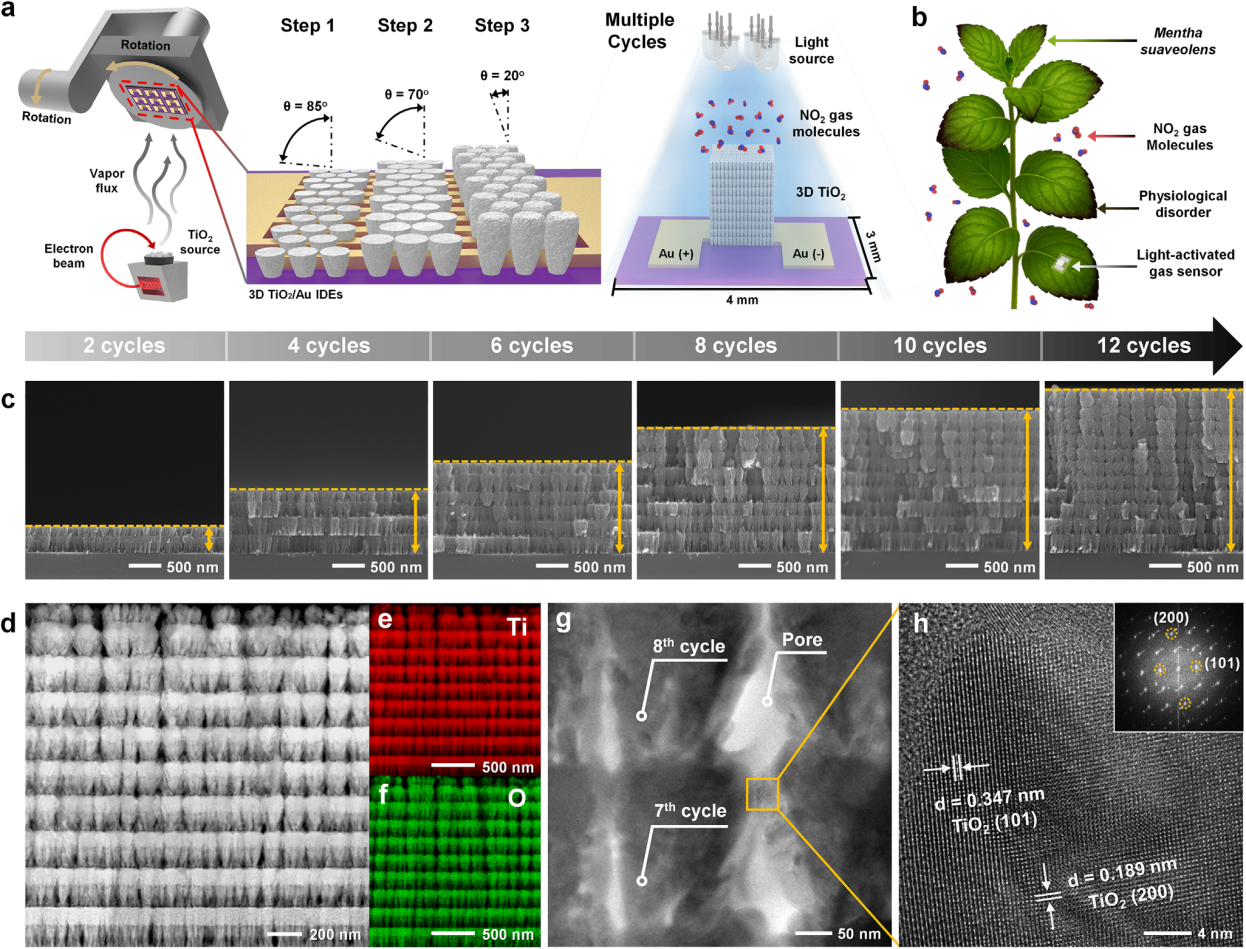
Fabrication and characterization of scalable 3D TiO_2_ nanoarchitectures. a) Schematic of the fabrication process for highly aligned 3D TiO_2_ nanoarchitectures. b) Schematic showing *Mentha suaveolens* under NO_2_‐induced physiological stress, integrated with a scalable NO_2_ gas sensor integrated on the leaf. c) Cross‐sectional SEM images of 3D TiO_2_ nanoarchitectures with varying numbers of deposition cycles: 2, 4, 6, 8, 10, and 12. d) Cross‐sectional STEM image of 3D TiO_2_. EDS mapping of e) Ti and f) O. HR‐TEM images of g) 3D TiO_2_ and h) TiO_2_. Inset in (h) shows the FFT image of TiO_2_.

Developing ambient‐compatible gas sensors with minimal energy requirements remains a key challenge in advancing next‐generation environmental electronics, especially for applications involving biological interfaces. To tackle this, we constructed a highly ordered, porous TiO_2_ framework using a stepwise GLAD technique. This vapor‐phase nanofabrication approach enabled the formation of vertically aligned 3D TiO_2_ architectures with precise geometrical control on wafer‐scale substrates (**Figure** **1**a). Sequential oblique‐angle depositions at variable incident angles, combined with continuous substrate rotation, induced self‐shadowing effects that directed anisotropic growth and the formation of interconnected nanochannels.^[^ ^33^ ^]^ This tailored 3D morphology provides both structural and functional benefits. Its periodicity and high porosity promote broadband light scattering and internal photon trapping, prolonging photon residence time within the material.^[^ ^30^ ^]^ Simultaneously, vertically aligned voids serve as continuous gas diffusion pathways, ensuring uniform NO_2_ penetration throughout the sensing volume.^[^ ^34^, ^35^ ^]^ These design features collectively facilitate efficient photoactivation under ambient light, eliminating the need for thermal assistance. Furthermore, the sensor's compact size (≈3 mm × 4 mm) and low weight (≈0.02 g) offer a practical advantage for deployment on thermally sensitive or soft substrates, such as plant leaves (Figure 1b).

The 3D porous structure was tuned from 2 to 12 deposition cycles, enabling programmable control over film thickness (≈400 nm to ≈2.5 µm), porosity (32.9–45.3%), and optical density (Figure 1c; Figure S1, Supporting Information). Cross‐sectional scanning electron microscopy (SEM) and high‐angle annular dark‐field scanning transmission electron microscopy (HAADF‐STEM) revealed well‐defined vertical formation, with homogeneous elemental distribution of Ti and O (Figure 1d–f). High‐resolution transmission electron microscopy (HR‐TEM) confirmed preserved anatase crystallinity (JCPDS card no. 21–1272) in the 10‐cycle 3D TiO_2_ nanoarchitecture (denoted as “3D TiO_2_”), with measured lattice spacings of d_101_ = 0.347 nm and d_200_ = 0.189 nm. Fast Fourier transform (FFT) analysis verified long‐range structural order across the porous network (Figure 1g,h).

The resulting highly periodic framework demonstrates excellent physical robustness, retains its hierarchical morphology over successive deposition cycles, and maintains high optical responsiveness essential for ambient‐light operation. Although the process includes high‐temperature annealing (500 °C), the GLAD method remains broadly compatible with standard wafer‐scale microfabrication workflows. With appropriate consideration of interconnect materials and thermal budgets, it can also be adapted for complementary metal‐oxide‐semiconductor (CMOS) integration. This compatibility supports further 3D integration into smart sensor systems, offering a scalable pathway toward optoelectronic sensor platforms embedded within biological environments.^[^ ^36^, ^37^ ^]^

### 2.2 Tunable Optical Properties via Highly Periodic Photonic Structuring

**Figure 2:**
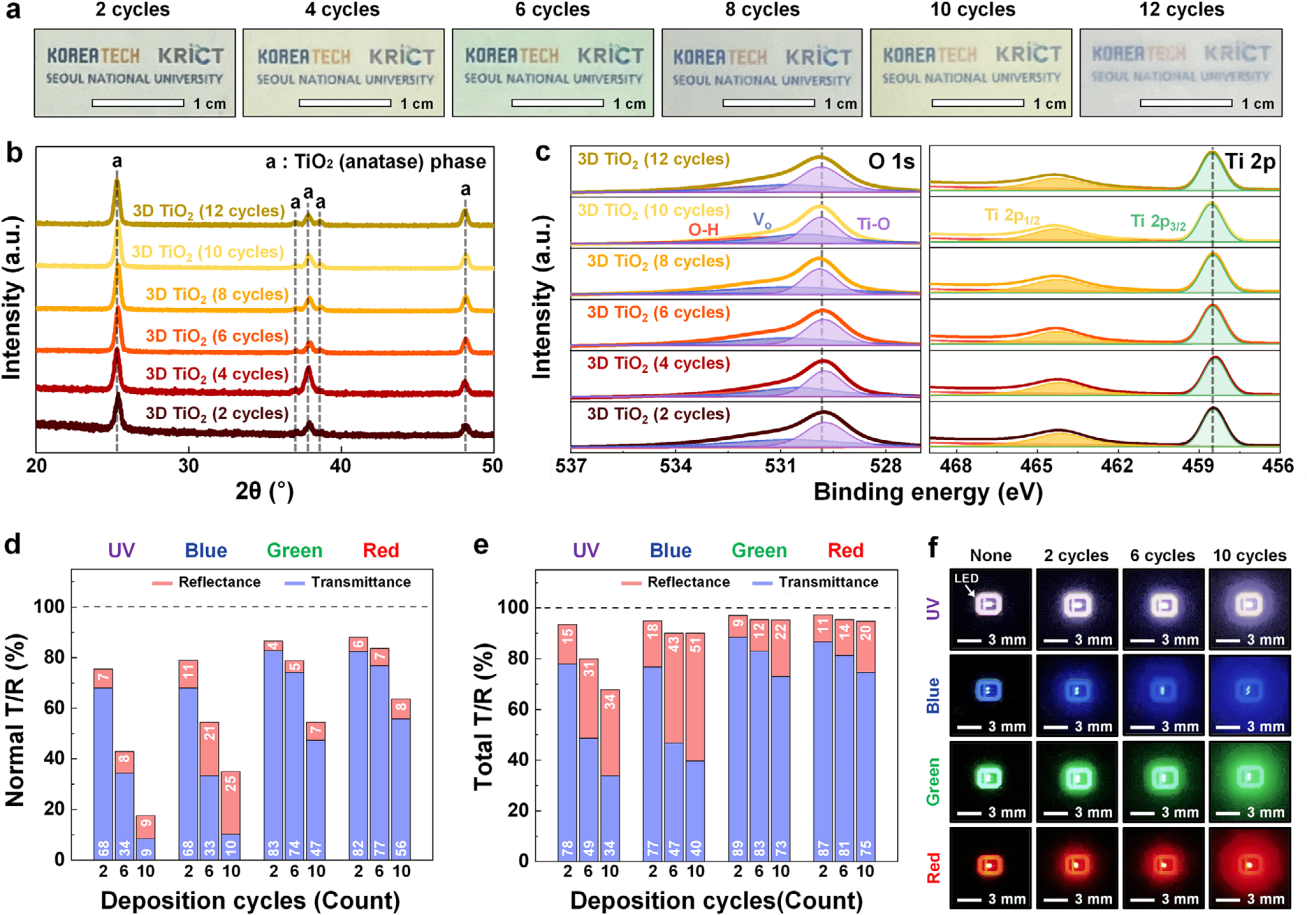
Cycle‐dependent optical scattering and diffusion in 3D TiO_2_ nanoarchitectures. a) Photographs of 3D TiO_2_ nanoarchitectures on a glass substrate with increasing the number of deposition cycles from 2 to 12. b) XRD patterns and c) XPS spectra of 3D TiO_2_ with 2 to 12 cyclic depositions. Comparison of d) normal and e) total T/R spectra of 2‐, 4‐, and 6‐cycle 3D TiO_2_ nanoarchitectures. f) Photographs of UV, blue, green, and red point‐source LEDs covered by 3D TiO_2_ nanoarchitectures with 0 (none), 2, 6, and 10 cyclic depositions.

The optical characteristics of the 3D TiO_2_ architecture were systematically modulated through cycle‐by‐cycle geometric tuning, which governed both the light‐handling capabilities and visual transparency of the material. As shown in **Figure** **2**a, increasing the number of deposition cycles from 2 to 12 transformed the films from transparent with a bluish structural color—arising from periodicity—to progressively more translucent, indicating increased internal light scattering without a significant change in thickness. This transition is attributed to increased multiple scattering within the porous structure, enabling effective manipulation of incident light across a broad spectral range.^[^ ^38^ ^]^ Despite the increased optical complexity, structural analyses confirmed that the underlying material integrity remained intact. X‐ray diffraction (XRD) patterns consistently showed the anatase phase across all cycle counts (Figure 2b), while X‐ray photoelectron spectroscopy (XPS) revealed stable Ti^4+^ oxidation states and uniform surface chemistry, with no evidence of defect accumulation or stoichiometric deviation (Figure 2c).

Quantitative optical analysis was performed to evaluate broadband scattering behavior. Normal transmittance and reflectance (T/R) spectra showed a monotonic decrease in transmittance and a corresponding increase in reflectance with additional cycling, spanning the UV, blue, green, and red regions (Figure 2d). Total T/R spectra confirmed that the highly periodic structures functioned as effective broadband optical diffusers, capable of laterally redistributing incoming photons across the film volume (Figure 2e). The gradually increasing difference between normal and total T/R values further supported the tunable optical properties of the 3D TiO_2_ nanoarchitectures (Figure S2, Supporting Information). This behavior was visually demonstrated through UV and RGB LED coverage experiments (Figure 2f): when placed atop point‐source LEDs, the films transformed confined emissions into spatially diffused glows—indicative of lateral photon redistribution through internal scattering. Notably, the 10‐cycle structure exhibited the most pronounced light diffusion effect, effectively converting narrow‐angle emission into broad‐area illumination. This highlights the potential of the 3D TiO_2_ framework to function as a photonic light‐spreading medium, particularly useful for achieving spatially uniform photoactivation in gas sensing applications.

Importantly, this broadband light‐scattering capability was achieved without increasing thermal load or requiring photonic crystals, making it especially well‐suited for compact, low‐power devices. The ability to tune both optical and physical parameters through deposition cycles provides a versatile and scalable design strategy for application‐specific spectral engineering, a crucial advantage for sensors operating in dynamic or light‐limited environments such as beneath foliage, on human skin, or within enclosed smart farming systems.

### 2.3 Photoactivated NO_2_ Detection with High Sensitivity, Selectivity, and Environmental Robustness

**Figure 3:**
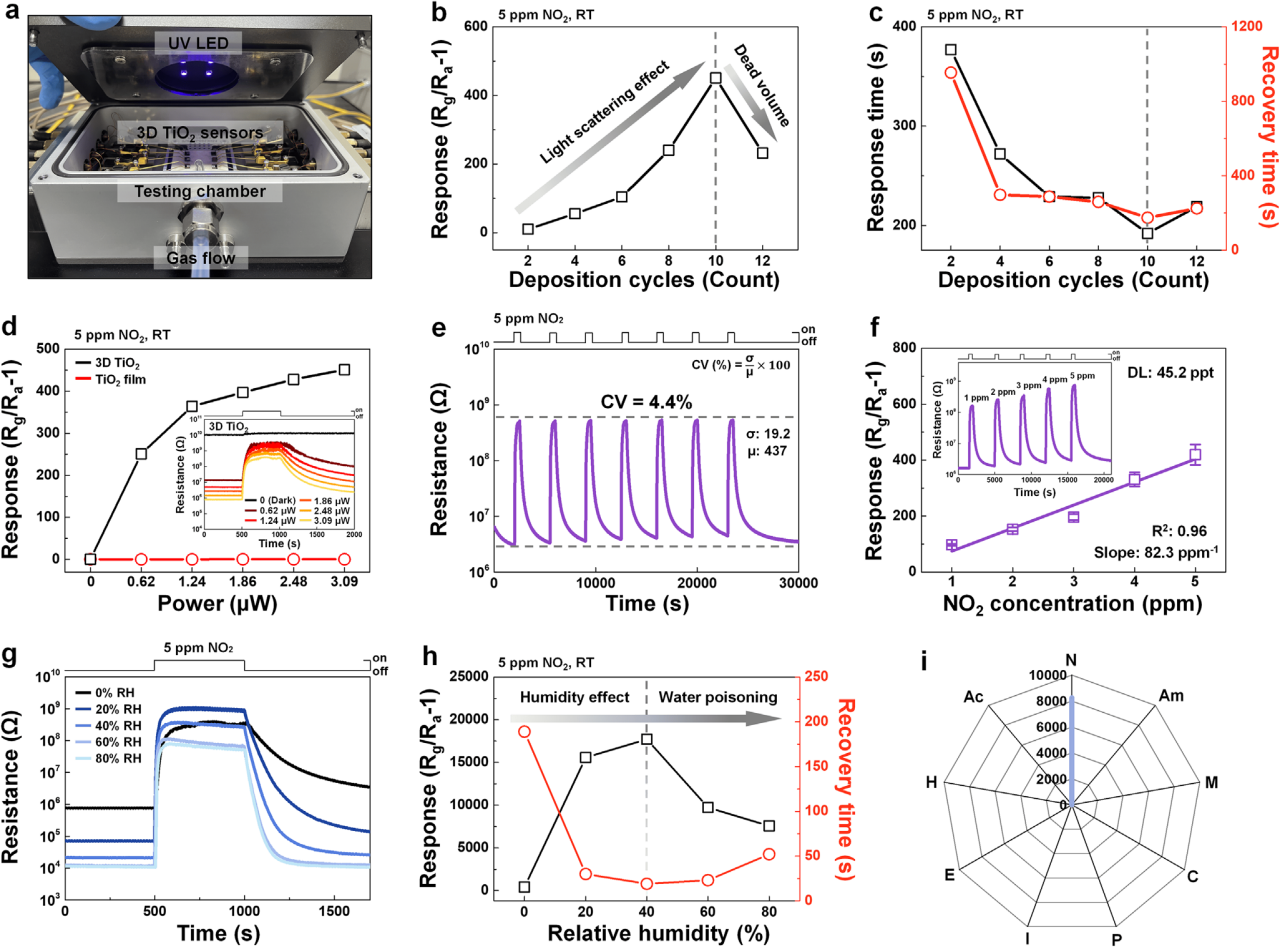
Outstanding performance of 3D TiO_2_ nanoarchitectures. a) Photograph of the experimental setup for chemiresistive gas sensing under UV LED illumination. b,c) Responses and response/recovery times of 3D TiO_2_ sensors with 2, 4, 6, 8, 10, and 12 cycles exposed to 5 ppm NO_2_. d) UV power‐dependent responses of the 3D TiO_2_ and TiO_2_ film exposed to 5 ppm NO_2_. Inset in (d) shows the corresponding resistance curves of 3D TiO_2_. e) Cyclic resistance curves of the 3D TiO_2_ under UV illumination exposed to 5 ppm NO_2_. f) Linear fit of the responses of the 3D TiO_2_ as a function of NO_2_ concentration (1–5 ppm). Inset in (f) shows the corresponding resistance curves. g,h) Resistance curves, responses, and recovery times of 3D TiO_2_ exposed to 5 ppm NO_2_ under RH of 0, 20, 40, 60, and 80%. i) Response radar plots of the 3D TiO_2_ exposed to 5 ppm NO_2_ and 50 ppm of eight interfering gases at 80% RH (N: nitrogen dioxide, Am: ammonia, M: methane, C: carbon monoxide, P: propane, I: isoprene, E: ethanol, H: hydrogen, and Ac: acetone).

To evaluate the sensing capabilities of the 3D TiO_2_ nanoarchitecture, its chemiresistive response to NO_2_ was examined under photoactivation at room temperature (**Figure** **3**a; Figure S3, Supporting Information). All measurements were performed under light illumination without external heating, confirming the thermally passive operation of the sensor. Given that applied voltage influences charge transport in semiconducting oxides, its effect on sensing performance was further investigated. Transitions between Ohmic behavior and space‐charge‐limited current (SCLC) or Poole–Frenkel (PF) emission can modulate free carrier density and interfacial charge dynamics, thereby affecting sensitivity and response kinetics.^[^ ^39^, ^40^, ^41^ ^]^ To investigate this, the 8‐cycle 3D TiO_2_ sensor was exposed to 5 ppm NO_2_ under UV illumination at varying bias voltages (5, 10, 20, and 30 V) (Figure S4, Supporting Information). All gas sensing properties—including gas response (defined as (*R_g_* − *R_a_*)/*R_a_*, where *R_g_* and *R_a_* represent the electrical resistance in the target gas and air, respectively), response time (*t*_90%_, air‐to‐gas), and recovery time (*t*_90%_, gas‐to‐air)—exhibited voltage‐dependent enhancement without thermal effects (Figure S5, Supporting Information), as discussed in a later section.

Structural optimization significantly influenced sensing performance. When exposed to 5 ppm NO_2_, the gas response increased with cyclic deposition count, peaking at ≈451 for 10 cycles before slightly declining at 12 cycles (Figure 3b; Figure S6, Supporting Information). This response profile reflects a critical trade‐off between enhanced light scattering and extended carrier pathways, where an intermediate cycle count achieves an optimal balance among surface area, optical absorption, and charge transport. Dynamic response and recovery times further highlight this optimal structure (Figure 3c). 3D TiO_2_ achieved the fastest response (≈192 s) and recovery (≈175 s), significantly outperforming thinner films, which exhibited prolonged recovery due to insufficient light trapping and slower gas diffusion. These trends confirm that the highly ordered, porous geometry not only enhances photon harvesting but also facilitates faster desorption kinetics and electronic reset.

To evaluate the geometric advantages of our 3D TiO_2_ structure, a conventional planar TiO_2_ film was fabricated and exposed to 5 ppm NO_2_ under both heating and UV illumination (Figures S7 and S8, Supporting Information). The planar sensor exhibited its highest response at 200 °C, with all sensing parameters outperforming those under UV activation. In contrast, 3D TiO_2_ exhibited superior sensing performance under UV illumination compared to thermal activation at 200 °C, achieving a 21.9‐fold signal increase, with reductions of 40.4% and 67.7% in response and recovery times, respectively (Figure S9, Supporting Information). This advantage of the 3D configuration was further corroborated by power‐dependent UV response comparisons between the 3D TiO_2_ and planar TiO_2_ film (Figure 3d; Figure S10, Supporting Information). 3D TiO_2_ exhibited a steeper response slope and an exponential decrease in recovery time with increasing UV intensity, indicating a more efficient conversion of optical energy into chemical sensitivity (Figure S11, Supporting Information). Figure 3e presents repeated NO_2_ exposure tests, demonstrating stable and reproducible response kinetics over seven cycles with a coefficient of variation (CV) of 4.4% (Figure S12, Supporting Information), highlighting the robust sensing performance compared to commercial gas sensors.^[^ ^42^, ^43^ ^]^ Moreover, the concentration‐dependent response to 1–5 ppm NO_2_ exhibited excellent linearity, yielding an extremely low theoretical detection limit (DL) of ≈45.2 ppt (Figure 3f), highlighting the sensor's suitability for low‐concentration NO_2_ monitoring.

The environmental stability of the device was assessed under varying relative humidity (RH) levels (0–80% RH, Figure 3g). The baseline resistance gradually decreased due to hydroxyl group formation on the TiO_2_ surface, while sensitivity increased—contrary to many oxide‐based sensors that typically degrade under humid conditions.^[^ ^44^, ^45^, ^46^, ^47^ ^]^ Notably, the response increased up to ≈40% RH due to hydroxyl‐assisted adsorption, then declined at higher RH levels owing to multilayer water formation and active site passivation (Figure 3h).^[^ ^29^ ^]^ The response and recovery times revealed similar trends, as discussed in a later section (Figure S13, Supporting Information). To confirm the sensor's robustness at high humidity (80% RH), additional gas measurements were conducted using multiple pulses of 5 ppm NO_2_ (Figure S14, Supporting Information) and varying concentrations (Figure S15, Supporting Information), showing strong performance with a low CV (8.5%) and an ultralow DL (≈66.3 ppq). These results suggest that the porous 3D TiO_2_ architecture inherently mitigates moisture‐induced suppression and maintains superior performance under ambient conditions, indicating strong potential for application in dynamically humid environments such as agricultural settings.

Selective NO_2_ recognition was confirmed by testing the device against eight common competing gases in agricultural systems (50 ppm NH_3_, CH_4_, CO, C_3_H_8_, C_5_H_8_, C_2_H_5_OH, H_2_, and CH_3_COCH_3_) in dry (Figures S16 and S17, Supporting Information) and 80% RH conditions (Figure 3i; Figure S18, Supporting Information). Because NO_2_ often coexists with other species in real environments, cross‐sensitivity was further evaluated by comparing responses to eighteen gas mixtures (eight NO_2_‐containing mixtures and ten interferent‐only mixtures) (Figure S19, Supporting Information). Notably, the 3D TiO_2_ sensor exhibited negligible interference from coexisting gases, underscoring robust NO_2_ detection under mixed‐gas conditions. These detection capabilities toward NO_2_ stem from room temperature photoactivation, which modulates adsorbed oxygen species and charge transfer on the surface.^[^ ^30^ ^]^ Under LED illumination, pre‐adsorbed oxygen species (O_2(ads)_^−^) recombine with photogenerated holes and subsequently desorb from the TiO_2_ surface.^[^ ^48^, ^49^, ^50^ ^]^ Meanwhile, photogenerated electrons react with oxygen molecules to generate weakly bound photogenerated oxygen ions (O_2(photo)_^−^).^[^ ^51^ ^]^ Given these competing processes, desorption of O_2(ads)_^−^ dominates over adsorption of O_2(photo)_^−^, providing reactive sites and releasing trapped electrons to TiO_2_, followed by reducing the resistance (Figure 1d; Figure S10, Supporting Information).^[^ ^30^, ^49^ ^]^ When interacting with O_2(photo)_^−^, oxidizing gases such as NO_2_ that extract electrons exhibit stronger responses than reducing gases that release electrons, owing to the excess photogenerated electrons trapped within TiO_2_, thereby resulting in high selectivity.

**Figure 4:**
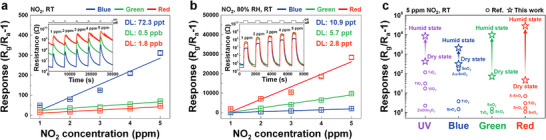
Linear fit of the responses of 3D TiO_2_ exposed to different NO_2_ concentrations under RGB LED illuminations. Insets in (a) and (b) show corresponding resistance curves. c) Comparison of 5 ppm NO_2_ sensing performance under UV and RGB LED illumination with state‐of‐the‐art photoactivated gas sensors.

Despite the wide bandgap of anatase TiO_2_ (≈3.2 eV), the sensor exhibited distinct photoresponses even under sub‐bandgap visible light illumination (**Figure** **4**a; Figure S20, Supporting Information).^[^ ^52^ ^]^ The humidity effect revealed enhanced responses with faster response/recovery times in 80% RH conditions (Figure 4b; Figure S21, Supporting Information). Moreover, all sensing performance indicated that a lower baseline resistance resulted in a higher response. The increase in photogenerated electrons under illumination reduced baseline resistance and promoted defect‐assisted charge transfer, resulting in enhanced NO_2_ detection. These findings suggest that the visible‐light sensitivity arises from the synergistic interplay of photon scattering, defect‐state absorption (e.g., Ti^3^⁺ and oxygen vacancies), and nanoconfinement‐enhanced carrier generation at low photon energies,^[^ ^53^, ^54^ ^]^ thereby enabling broad spectral responsiveness. Overall, the 3D TiO_2_ sensor substantially enhanced the sensing performance, including response, DL, and response/recovery times, even within the mid‐gap state, compared with state‐of‐the‐art light‐activated gas sensors across broad spectral ranges (Figure 4c; Table S1, Supporting Information).^[^ ^30^, ^47^, ^55^, ^56^, ^57^, ^58^ ^]^

Collectively, these results demonstrate that the 3D TiO_2_ nanoarchitecture is a structurally optimized platform for photoactivated NO_2_ sensing with high selectivity, humidity resilience, and broad spectral responsiveness. This tailored approach eliminates the need for dedicated light sources or thermal excitation, thereby ensuring energy‐efficient operation and making it highly promising for deployment in real‐world ambient and biologically integrated systems.

### 2.4 Mechanistic Elucidation of Light‐Driven Charge Transport and Humidity Resilience

**Figure 5:**
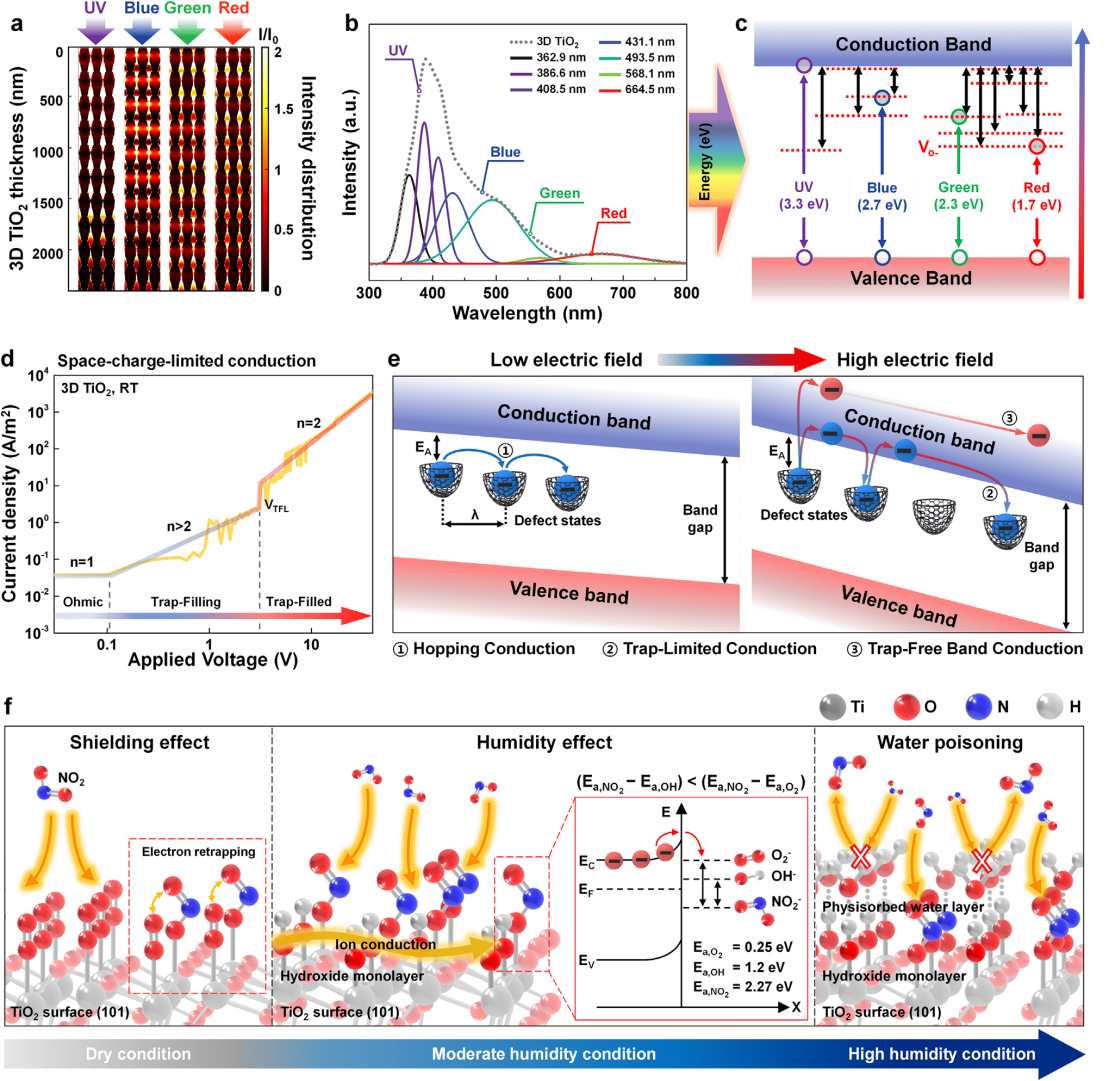
Mechanistic elucidation of the enhanced performance. a) Calculated E‐field intensity distributions of 3D TiO_2_ under illumination of various LEDs: UV (365 nm), blue (470 nm), green (532 nm), and red (680 nm). b) Photoluminescence spectra of 3D TiO_2_. c) Reconstructed band diagram of 3D TiO_2_ derived from the photoluminescence data shown in (B). d) Electronic band diagram under low and high E‐fields. e) log J versus log V characteristics of 3D TiO_2_ under UV illumination for SCLC analysis. f) Schematic showing the humidity‐dependent conduction mechanism on the TiO_2_ surface at room temperature and the energy band diagram of the TiO_2_ surface with relative adsorbate levels of NO_2_, OH, and O_2_. The ordering of these levels reflects the free‐space electron affinities of the corresponding molecules.

To elucidate the mechanisms underlying light‐activated NO_2_ sensing, we performed a comprehensive mechanistic analysis integrating optical simulations, spectroscopic characterization, and electrical transport modeling. Finite‐difference time‐domain simulations under varied illumination conditions (UV, blue, green, red) revealed that the 3D TiO_2_ nanoarchitecture exhibits distinct wavelength‐dependent behavior (**Figure** **5**a). Under UV excitation, absorption is predominantly localized near the surface, whereas under visible light, electric field (E‐field) enhancement occurs along interfaces between nanopillars and voids. This interfacial field intensification arises from strong scattering and internal reflection, which extend the effective optical path length and promote photon–matter interactions even at sub‐bandgap photon energies. Photoluminescence spectroscopy confirmed broadband emission in the visible region (Figure 5b), consistent with mid‐gap states derived from oxygen vacancies and Ti^3^⁺ species. These defect states enable visible‐light‐induced excitation, as illustrated in the band diagram (Figure 5c). While UV light promotes direct transitions across the anatase bandgap (≈3.2 eV), blue, green, and red photons activate electrons from mid‐gap defect states into the conduction band. This mechanism explains the measurable photoresponse of the device under low‐energy visible light, despite the UV‐active nature of intrinsic TiO_2_.

To investigate how the applied bias affects the sensitivity of the 3D TiO_2_ architecture, we measured the current density–voltage (*J–V*) characteristics of the device. The typical *J–V* curve was replotted on a double‐logarithmic scale to elucidate the electrical transport behavior of the 3D TiO_2_ architecture, as shown in Figure 5d, with a schematic illustration of the corresponding conduction mechanisms in Figure 5e. The 3D TiO_2_ architecture exhibits linear Ohmic conduction with a slope of 1, indicating free carrier drift.^[^ ^40^ ^]^ As the applied bias increases, the device transitions to a trap‐limited space‐charge‐limited conduction (SCLC) regime, where injected carriers occupy localized states. Under a high applied bias of above 2.1 V, the 3D TiO_2_ architecture exhibits trap‐free band conduction with a slope of 2. This result suggests that the highly ordered, porous TiO_2_ framework, with its superior trap‐passivation capability, facilitates efficient carrier transport (trap‐free conduction) under relatively low applied bias. In contrast, the planar TiO_2_ film still exhibits trap‐limited SCLC even at a high applied bias (40 V) owing to incomplete trap filling (Figures S22 and S23, Supporting Information).^[^ ^59^, ^60^ ^]^ Notably, compared to the planar TiO_2_ film, PF conduction also appears in the 3D TiO_2_ architecture over a wide bias range, enabling the excitation of trapped electrons (Figure S24, Supporting Information). These excited electrons contribute to the conduction process and accelerate the desorption of gas molecules from the semiconductor surface,^[^ ^41^ ^]^ leading to faster recovery and higher sensitivity in the 3D TiO_2_ architecture.

Humidity responsiveness was further examined through surface interaction modeling (Figure 5f). In dry air, NO_2_ interacts with lattice oxygen and surface hydroxyls, generating charge transfer that modulates conductivity.^[^ ^29^, ^61^ ^]^ At moderate humidity (≈40% RH), additional hydroxyl groups facilitate NO_2_ adsorption and enhance sensitivity.^[^ ^62^, ^63^, ^64^, ^65^, ^66^ ^]^ However, at higher humidity (> 60% RH), a physisorbed water layer forms, impeding gas access and passivating reactive sites, thereby reducing the signal amplitude and prolonging recovery time.^[^ ^67^ ^]^ This transition highlights the dual role of water in oxide‐based sensors, where it can both promote and inhibit sensing.

Together, these findings reveal a coherent mechanism in which light‐scattering geometry, defect‐mediated sub‐bandgap excitation, and field‐assisted charge transport enable thermally passive, spectrally adaptive NO_2_ sensing with high selectivity and environmental stability. The integration of photonic and electronic functionalities within a porous crystalline matrix underpins the robustness and versatility of this platform in real‐world conditions.

### 2.5 Autonomous Plant‐Integrated Deployment for Smart Environmental Monitoring

**Figure 6:**
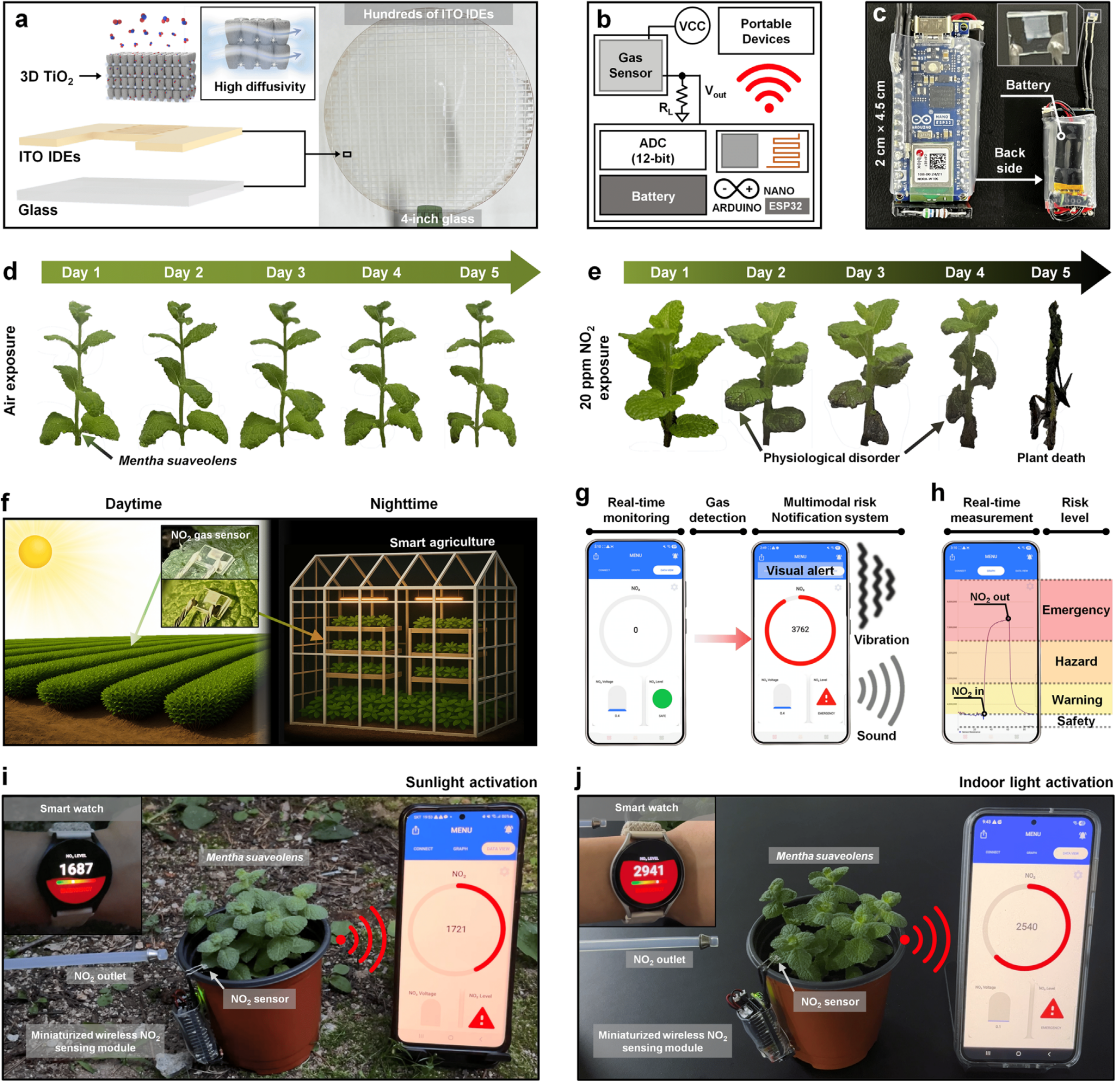
Autonomous plant‐integrated NO_2_ monitoring system. a) Exploded‐view schematic of transparent 3D TiO_2_/ITO IDEs and wafer‐scale fabrication photographs on 4‐inch glass substrates. b,c) Circuit diagram and photographs of a miniaturized wireless NO_2_ sensing module. Optical images of *Mentha suaveolens* over 5 days under d) ambient air and e) 20 ppm NO_2_ exposure. f) Schematic of an autonomous plant‐integrated NO_2_ monitoring system under sunlight and indoor illumination. g,h) User interface of the real‐time gas monitoring application and standard risk levels. Field demonstration of a real‐time NO_2_ sensing system under i) outdoor and j) indoor conditions.

The real‐world deployment of optoelectronic gas sensors has been limited by challenges related to power consumption, environmental durability, and biological compatibility. To address these challenges, we developed a fully operational, miniaturized NO_2_ monitoring platform capable of autonomous operation in plant‐associated environments. The system incorporates a wafer‐scale, transparent gas sensor fabricated using vertically aligned 3D TiO_2_ nanoarchitectures deposited on indium tin oxide (ITO) interdigitated electrodes (IDEs) patterned on glass substrates (**Figure** **6**a). Wafer‐scale uniformity was assessed across ten sensors at five wafer positions (top, bottom, left, right, center), yielding consistent responses with an inter‐device CV of 4.8% (Figure S25, Supporting Information). This transparent architecture facilitates direct optical access to the TiO_2_ active sites, enabling ambient light—whether solar or horticultural LED—to penetrate and drive the sensing process without thermal input. The porous 3D geometry simultaneously supports both gas permeation and photon trapping, providing structural advantages for both photoactivation and analyte accessibility. Moreover, this sensor fabrication is fully compatible with scalable, low‐temperature, and MEMS‐adaptable processes, supporting integration into large‐area, modular environmental monitoring systems. For portable operation, the sensor was integrated with a compact wireless module containing an ESP32 microcontroller, a rechargeable battery, and a Bluetooth module (Figure 6b,c). Housed within a 2 cm × 4.5 cm form factor, the module supports decentralized sensing without the need for external infrastructure. Notably, the micro‐watt‐level power consumption of the sensing device allows operation for over 200 days when powered by a commercially available 1000 mAh Li‐Po battery, demonstrating the device's long‐term applicability. The system can be noninvasively integrated into plant‐growing environments, such as potted plants or greenhouse trays, preserving both aesthetic and biological functions.

To evaluate biological relevance, *Mentha suaveolens* was exposed to 20 ppm NO_2_ over five days (Figure S26, Supporting Information). The control group, maintained under ambient air, retained a healthy morphology (Figure 6d), whereas the NO_2_‐exposed plants (Figure 6e) exhibited progressive chlorosis, tissue dehydration, and eventual collapse—confirming the direct phytotoxic effects of NO_2_ and validating the need for localized sensing.^[^ ^18^, ^19^, ^20^, ^64^ ^]^ Figure 6f illustrates a smart farm concept enabled by this sensor. In outdoor conditions, the device leverages solar illumination to drive sensing, whereas at night or indoors, horticultural LED lighting sustains operation. The sensor dynamically calibrates to ambient lighting conditions, ensuring stable performance across diurnal cycles and fluctuating environments. A dedicated mobile application enables real‐time user interaction via Bluetooth, issuing alerts through vibration, sound, and visual cues, along with corresponding risk levels when NO_2_ concentrations exceed safety thresholds (Figure 6g,h; Figure S27, Supporting Information). Resistance‐time plots and color‐coded risk indicators enable intuitive decision‐making and prompt environmental intervention. Notably, all processing is performed locally on the device, eliminating the need for cloud connectivity and ensuring a low‐latency, privacy‐preserving architecture for decentralized monitoring.

Field tests conducted by placing the sensor on plant leaves under both sunlight (Figure 6i) and indoor illumination (Figure 6j) confirmed operational robustness and signal reproducibility (Figure S28 and Videos S1–S4, Supporting Information). The sensor consistently responded to NO_2_ under ambient light with minimal signal drift and also demonstrated distinct gas response to seven types of commercial LEDs, further supporting its applicability across diverse real‐world illumination sources (Figure S29, Supporting Information). To the best of our knowledge, these results represent the first time‐resolved demonstration of nanomaterial‐based NO_2_ detection directly coupled with physiological plant responses in real‐world settings. This operational reliability highlights the potential of the platform for integration into smart farming systems, environmental safety infrastructure, and plant‐interfaced sensor networks.

## 3 Conclusion

We developed a scalable, light‐activated gas sensing platform based on wafer‐scale 3D TiO_2_ nanoarchitectures, enabling thermally passive and selective detection of NO_2_ under ambient and sub‐bandgap illumination. Through the precise geometric control of porosity and optical density, the sensor combines broadband light scattering, high surface accessibility, and defect‐assisted photoactivation to achieve an efficient chemiresistive response without external heating. This structural and optical tunability was directly correlated with enhanced sensitivity, faster response kinetics, and improved humidity tolerance—key performance metrics for real‐world environmental applications.

By integrating the sensing element with a wireless microcontroller and mobile interface, we demonstrated fully autonomous NO_2_ monitoring in both indoor and outdoor plant environments. Field deployment on *Mentha suaveolens* revealed a clear correlation between sensor output and visible physiological stress, confirming the ecological relevance and operational robustness of the system. The ability to operate under ambient light, including sunlight and indoor LED lighting, coupled with dynamic baseline adaptation and real‐time app‐based alerts, supports its use in decentralized environmental monitoring systems.

Beyond agricultural NO_2_ detection, the underlying platform offers a versatile foundation for light‐powered environmental sensing, with potential applications in greenhouse automation, urban air quality monitoring, and wearable interfaced electronics for plants or human skin.^[^ ^27^, ^68^, ^69^, ^70^ ^]^ Its compatibility with wafer‐scale microfabrication and CMOS‐compatible thermal budgets further facilitates integration with low‐power electronic modules, broadening its applicability across diverse environmental and biological sensing platforms. Future directions may include the incorporation of multimodal sensors (e.g., VOCs, humidity, temperature), as well as machine learning‐based signal interpretation for long‐term deployment in closed‐loop biosystems.

## 4 Experimental Section

### Preparation of 3D TiO_2_‐Based Gas Sensors

Au IDEs were fabricated using photolithography followed by electron beam deposition. SiO_2_/Si substrates were sequentially cleaned in an ultrasonic bath with acetone, ethanol, and deionized (DI) water, each for 5 min. A bottom photoresist layer (LOR 5A, MicroChem) was spin‐coated at 3000 rpm for 30 s and soft‐baked at 190 °C for 5 min. A top photoresist layer (AZ GXR 601, AZ Electronic Materials) was then applied under the same spin conditions and baked at 150 °C for 1 min. IDE patterns were defined using a mask aligner system (MDA‐400S, MIDAS System), followed by development in AZ 300MIF (MicroChemicals) and rinsing with DI water. After patterning, Au/Pt/Cr (100 nm/70 nm/30 nm) layers were deposited onto the patterned SiO_2_/Si substrates using an electron beam evaporator (EBX‐1000, ULVAC). The lift‐off process was performed using mr‐Rem 700 (micro resist technology), and the substrates were cleaned with ethanol and DI water. The resulting Au IDEs comprised 20 fingers with a spacing of 5 µm.

To control the thickness of 3D TiO_2_, TiO_2_ (99.99%, Taewon Scientific Co.) was sequentially deposited onto the Au IDEs using an electron beam evaporator (Korea Vacuum), while the substrate was repeatedly tilted at angles of 85°, 70°, and 20°, with a deposition rate of 3 Å s^−1^ and a rotation speed of 18 rpm. The final cycle was conducted only at 85° and 70°. A TiO_2_ film was also fabricated on a substrate positioned at the original angle (0°). All samples were then annealed at 500 °C for 1 h in ambient air to induce TiO_2_ crystallization.

### Characterizations

The cross‐sectional and top‐view morphologies of the 3D TiO_2_ nanoarchitectures and TiO_2_ film were examined using field‐emission scanning electron microscopy (FE‐SEM, JSM‐7610F‐Plus, JEOL) operated at an acceleration voltage of 15 kV and a working distance of 8 mm. The porosity ratio between TiO_2_ (white) and void (black) regions was quantified from top‐view SEM images converted to binary format using MATLAB R2023b (MathWorks, Natick, MA, USA). The detailed cross‐sectional morphology and crystallographic structure of the 3D TiO_2_ were characterized using transmission electron microscopy (TEM, Tecnai G2 F20, FEI) operated at an acceleration voltage of 200 kV. Additional crystallographic information was obtained in the 2θ range of 20–60° through XRD (Empyrean, PANalytical) using Cu‐Kα radiation (λ = 1.5418 Å), with a current of 30 mA and tube voltage of 40 kV. Chemical states were analyzed by XPS (AXIS SUPRA, KRATOS) using monochromated Al Kα radiation, with the binding energy scale referenced to the C 1s peak at 284.5 eV. Normal T/R spectra were acquired using a UV–vis system comprising a light source (AvaLight‐DHc), spectrometer (AvaSpec‐ULS2048CL‐EVO), and AvaSoft 8.16 software. Total T/R spectra were measured using a UV–vis–NIR spectrophotometer (V‐770, Jasco) and SpectraManager 2.5 software. PL spectra were obtained using a fluorescence spectrophotometer (F‐7000, Hitachi) equipped with a 150 W xenon lamp as the excitation source.

ln(J)–V characteristics were measured using a source meter (Keithley 237, KEITHLEY), a 12‐channel probe system (MPS6000, PHOCOS), a high‐density switch system (7001, KEITHLEY), and 237–7001 Multi Channel Measurement Software. ln(J/V)–E^1/2^ characteristics were analyzed using a motorized probe station (M7VC_Motorized, MSTECH) connected to a semiconductor parameter analyzer (E5270B, Keysight Technologies) and an LCR meter (E4980A, Keysight Technologies).

### Gas Sensing Measurement for Nine Gases

The gas‐sensing setup consisted of a compact probe chamber equipped with a 12‐channel probe system (MPS6000, PHOCOS) and a mass flow controller (PHOCOS) for precise regulation of gas concentration and RH. The dimensions of the mini probe chamber were 140 mm × 80 mm × 40 mm (width × length × height). Gas response measurements were performed for nine gases (NO_2_, NH_3_, CH_4_, CO, C_3_H_8_, C_5_H_8_, C_2_H_5_OH, H_2_, and CH_3_COCH_3_, all balanced with air, RIGAS) under DC bias voltages of 5, 10, 20, and 30 V, using a source meter (Keithley 238, KEITHLEY). After the sensor resistance (RS) stabilized to its baseline resistance in air, the target gases were introduced at a total flow rate of 1000 sccm using an automated gas delivery system. A high‐density switch system (7001, KEITHLEY) was used to enable precise, simultaneous resistance measurements across multiple sensors. Dynamic resistance changes were recorded at ≈1 s intervals using the 237–7001 Multi Channel Measurement Software.

### Calculations of Theoretical DL

The DL was calculated with a signal‐to‐noise ratio of 3, using the following equations:

(1) $$Vx^{2}=\sum {\left(y_{i}-y\right)}^{2}$$

(2) $$\mathrm{rms}\;=\sqrt{\frac{Vx^{2}}{N}}$$

(3) $$\mathrm{DL}=3\left(\frac{\mathrm{rms}}{\mathrm{slope}}\right)$$

Prior to target gas exposure, 10 consecutive resistance values were recorded to establish the sensor's baseline. These values, denoted as *y_i_*, represent the sensor response before gas injection and were used to calculate deviations from the mean response (*y*), obtained by fitting the data to a fifth‐order polynomial curve. The variance *Vx* ^2^ was calculated using Equation (1), and the root‐mean‐square (rms) noise was derived from Equation (2), where 𝑁 is the number of data points used in the fitting process. The DL for 3D TiO_2_ was then calculated for NO_2_ under both dry and 80% RH conditions using Equation (3).

### Fabrication of Autonomous Plant‐Integrated NO_2_ Monitoring System

An autonomous NO_2_ sensing system was developed for integration into plant‐associated environments. The platform utilizes an Arduino Nano ESP32 microcontroller, powered by two 3.7 V lithium‐polymer batteries connected in series (7.4 V total), supplying power to both the controller and sensor module. The sensing circuit includes a user‐adjustable load resistor (RL) to accommodate varying gas concentrations. The Arduino connects to a predefined Wi‐Fi router in the Station (STA) mode and functions as a local server. A client‐side mobile application communicates with the device via a fixed IP and port, receiving sensor ADC data at 1‐s intervals. The measured voltage signals were converted into RS based on the defined RL value and visualized in real time within the application.

Hazard levels were categorized into four stages (Levels 0–3) based on RS thresholds: SAFE (Level 0), WARNING (Level 1), HAZARD (Level 2), and EMERGENCY (Level 3). Alerts were triggered from Level 1 and escalated in intensity through smartphone audio alarms and smartwatch vibration feedback. A graphical arc progress indicator within the application interface conveys the severity of the detected risk.

### Statistical Analysis

The detection capability was assessed using a single device (n = 1), and wafer‐scale uniformity was evaluated across ten sensors (n = 10) fabricated on the 4‐inch wafer substrate. All cyclic measurement data were presented as mean and standard deviation, as well as CV. The response values of eighteen gas mixtures were normalized to the response toward 5 ppm NO_2_. Since the experiments focused on real‐time monitoring of plant health rather than population‐related inference, no inferential statistical tests (e.g., *t*‐test or ANOVA) were performed. Top‐view SEM images were converted to a binary image, and porosity was quantified using MATLAB R2023b (MathWorks, Natick, MA, USA).

## Conflict of Interest

The authors declare no conflict of interest.

## Supporting information



Supporting Information

Supplemental Video 1

Supplemental Video 2

Supplemental Video 3

Supplemental Video 4

## Data Availability

The data that support the findings of this study are available in the supplementary material of this article.
