# Supplementary material for: Scalable Photoactive NO2‐Sensing Framework for Plant Health Monitoring
Source: Adv Sci (Weinh). 2025 Nov 6;13(5):e18368. doi: 10.1002/advs.202518368 (PMC12850083; doi:10.1002/advs.202518368)
Supplement: Supplementary file 1 — Supporting Information [file ADVS-13-e18368-s004.docx]

Supporting Information

Scalable Photoactive NO_2_-Sensing Framework for Plant Health Monitoring

Yun-Haeng Cho, Kootak Hong, Jung Hwan Seo, Jae Han Chung, Jinho Lee, Sang-Hyeon Nam, Sunwoo Lee, Jeong-O Lee, Changui Ahn, Hyojung Kim, Jae Hyun Han, Gyu-Li Kim, Seong-Jun Ro, Jun Yeon Hwang, Hyeongyu Gim, Zion Park, Chil-Hyoung Lee, Dong-Su Kim, Kwangjae Lee*, Young-Seok Shim*, Jun Min Suh*, Donghwi Cho*


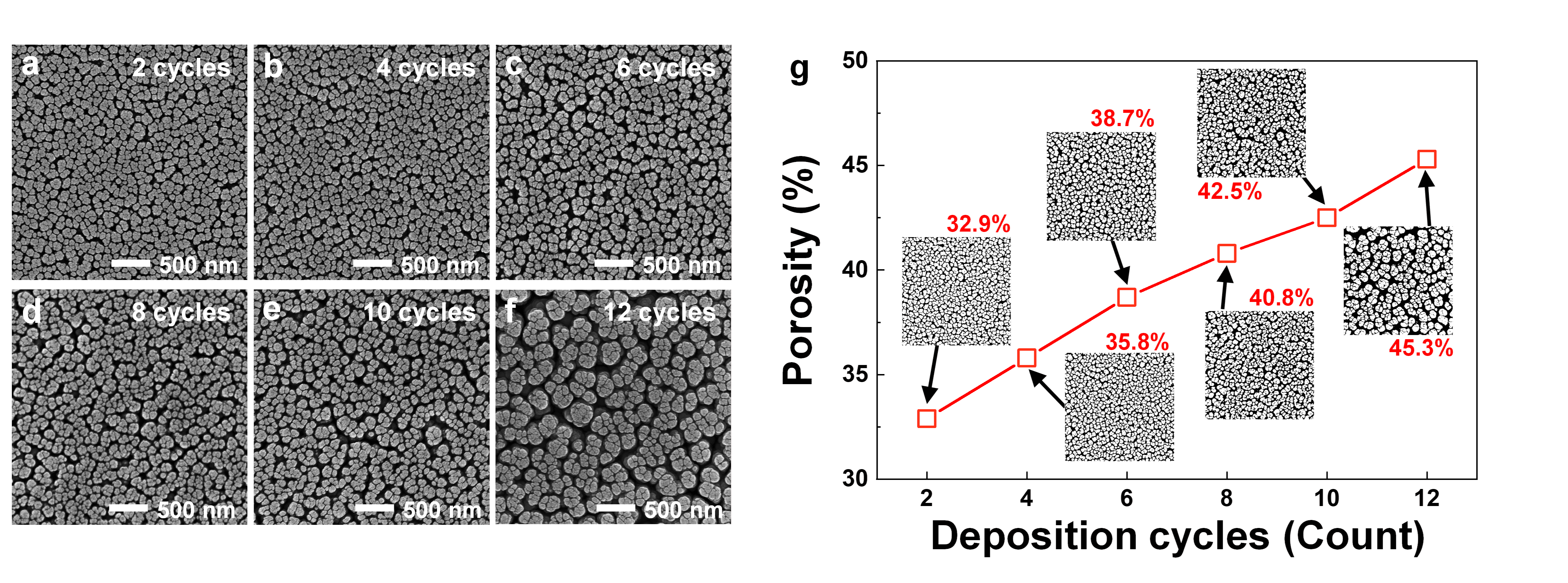


**Figure S1.** a–f) Top-view SEM images of 2-, 4-, 6-, 8-, 10-, and 12-cycle 3D TiO_2_. g) Calculated porosities of 2-, 4-, 6-, 8-, 10-, and 12-cycle 3D TiO_2_ processed in MATLAB. Inset displays the binary top-view SEM images obtained using MATLAB.


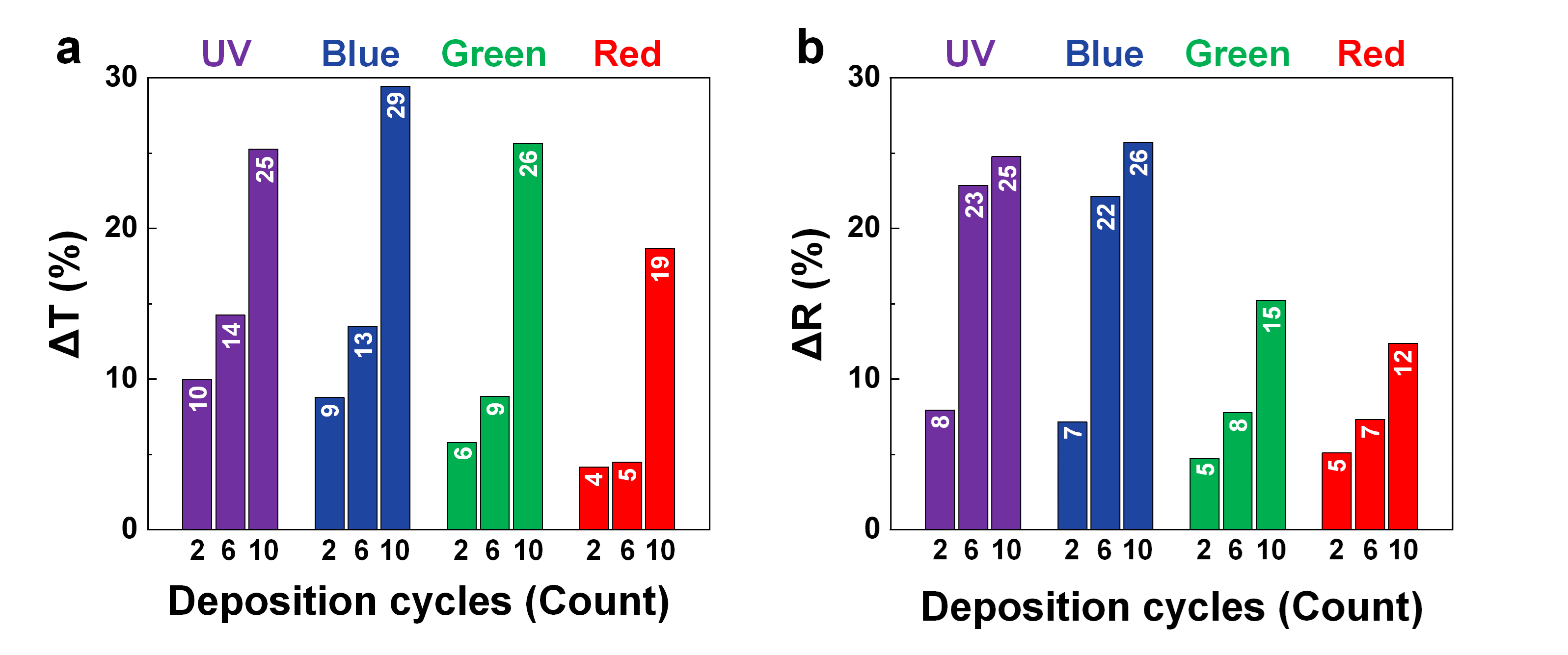


**Figure S2.** Comparison of a) total and b) normal T/R spectra of 2-, 6-, and 10-cycle 3D TiO_2_. $\Delta$T and R denote the differences between the total and normal values.


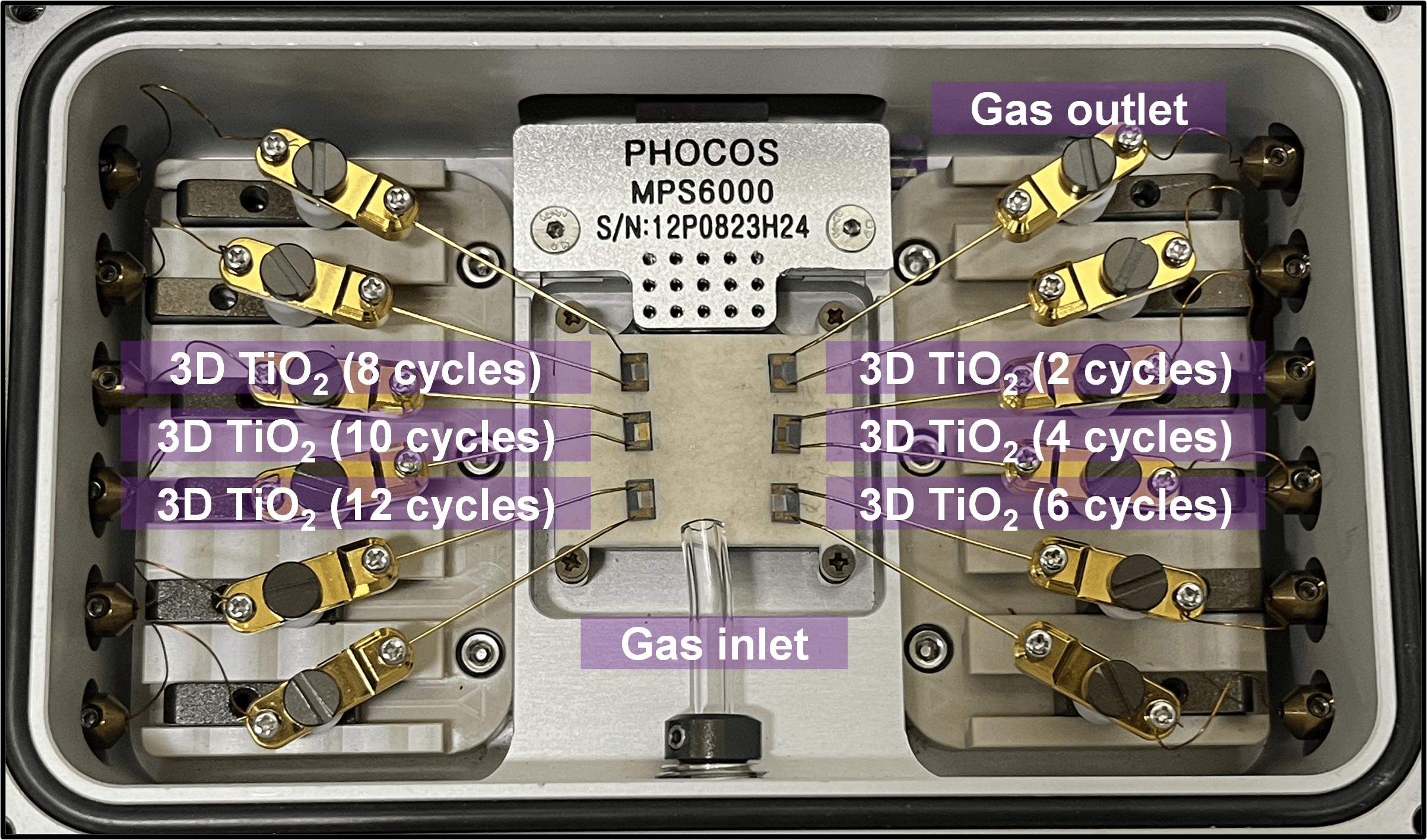


**Figure S3.** Photograph of the 12-channel probe system.


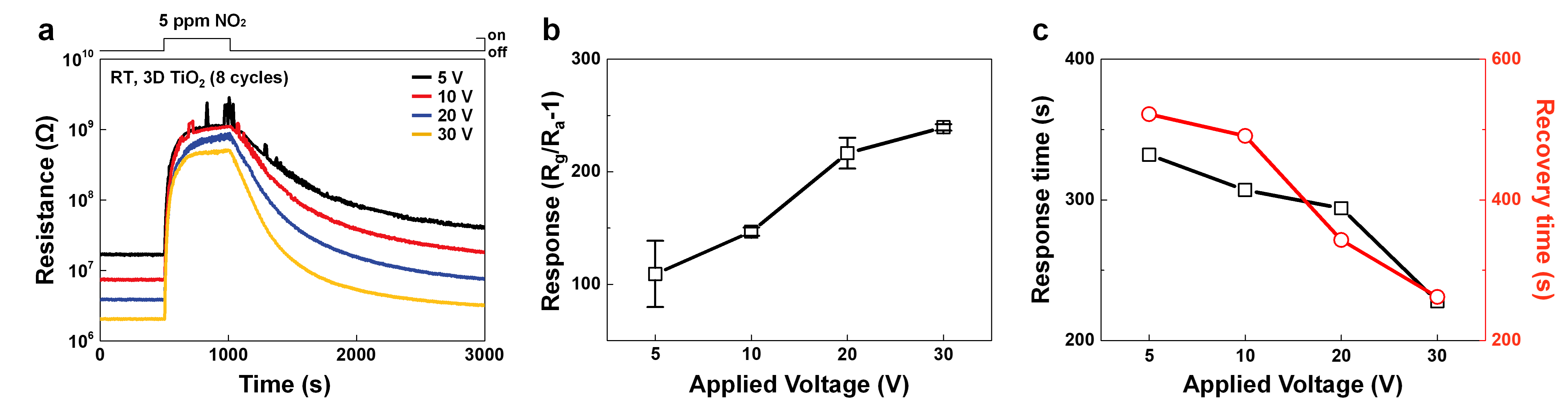


**Figure S4.** a) Resistance curves, b) responses, and c) response/recovery times of 3D TiO_2_ (8 cycles) exposed to 5 ppm NO_2_ under UV illumination at applied voltages of 5, 10, 20, and 30 V.


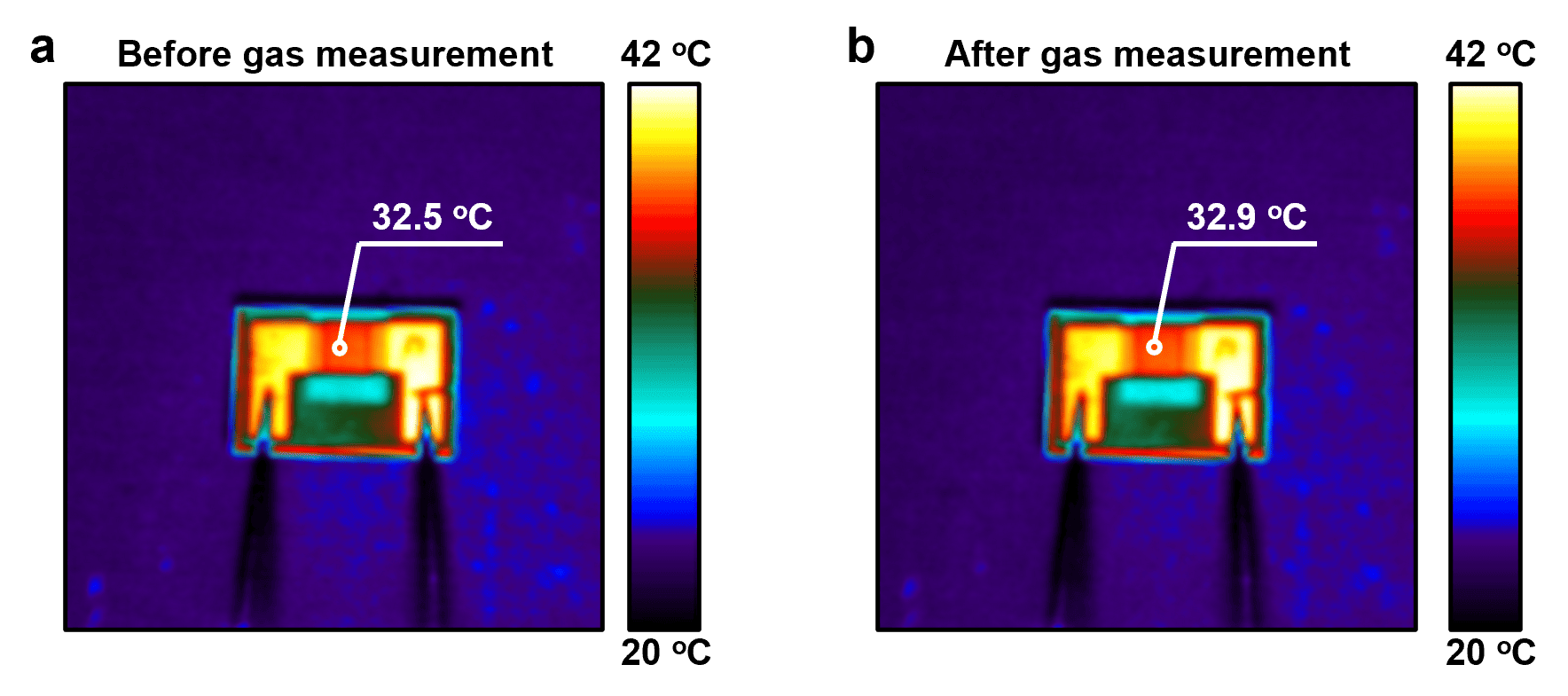


**Figure S5.** Thermographic images of 3D TiO_2_ (8 cycles) a) before and b) after 3 h of gas exposure to 5 ppm NO_2_ under UV illumination.


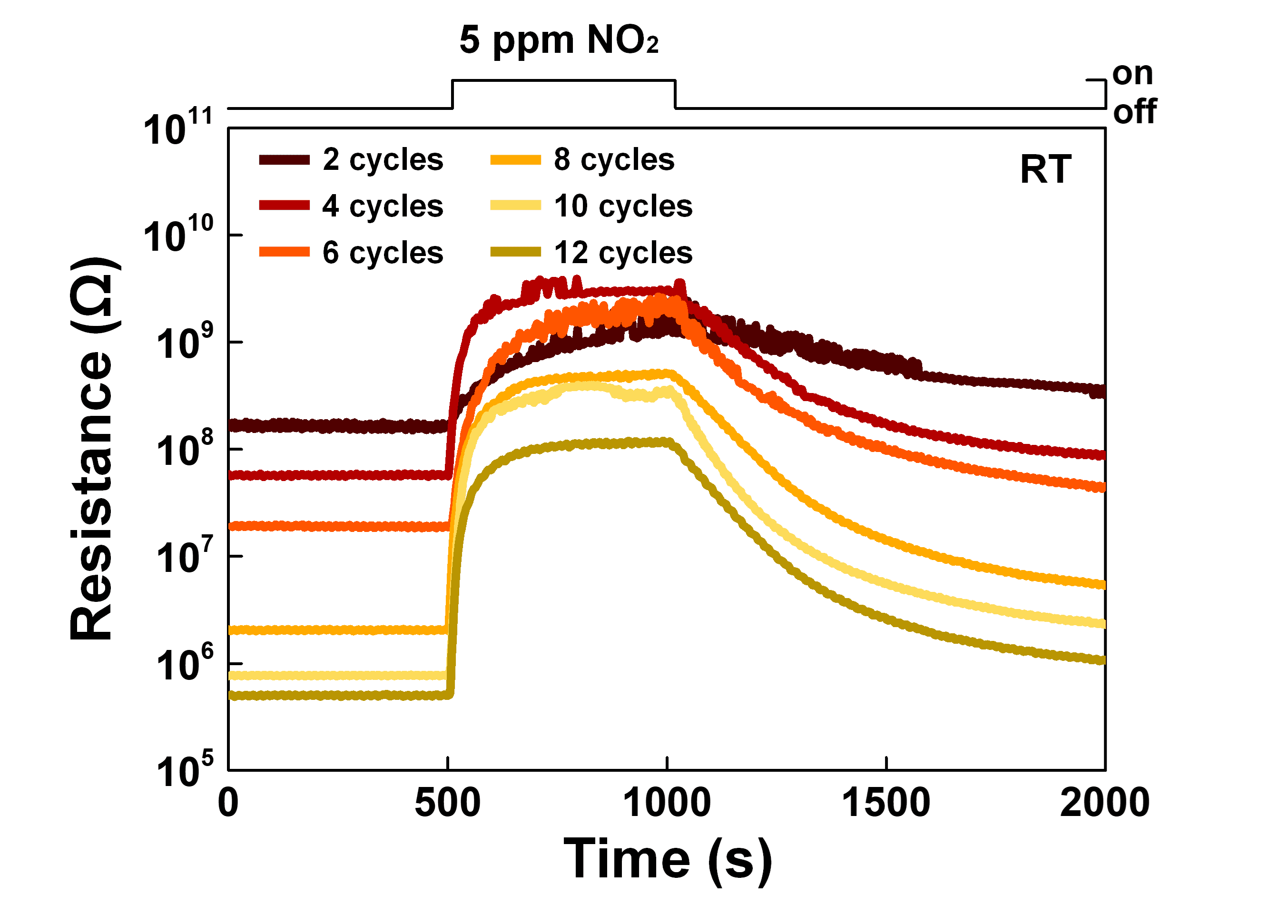


**Figure S6.** Resistance curves of 2-, 4-, 6-, 8-, 10-, and 12-cycle 3D TiO_2_ exposed to 5 ppm NO_2_ under UV illumination.


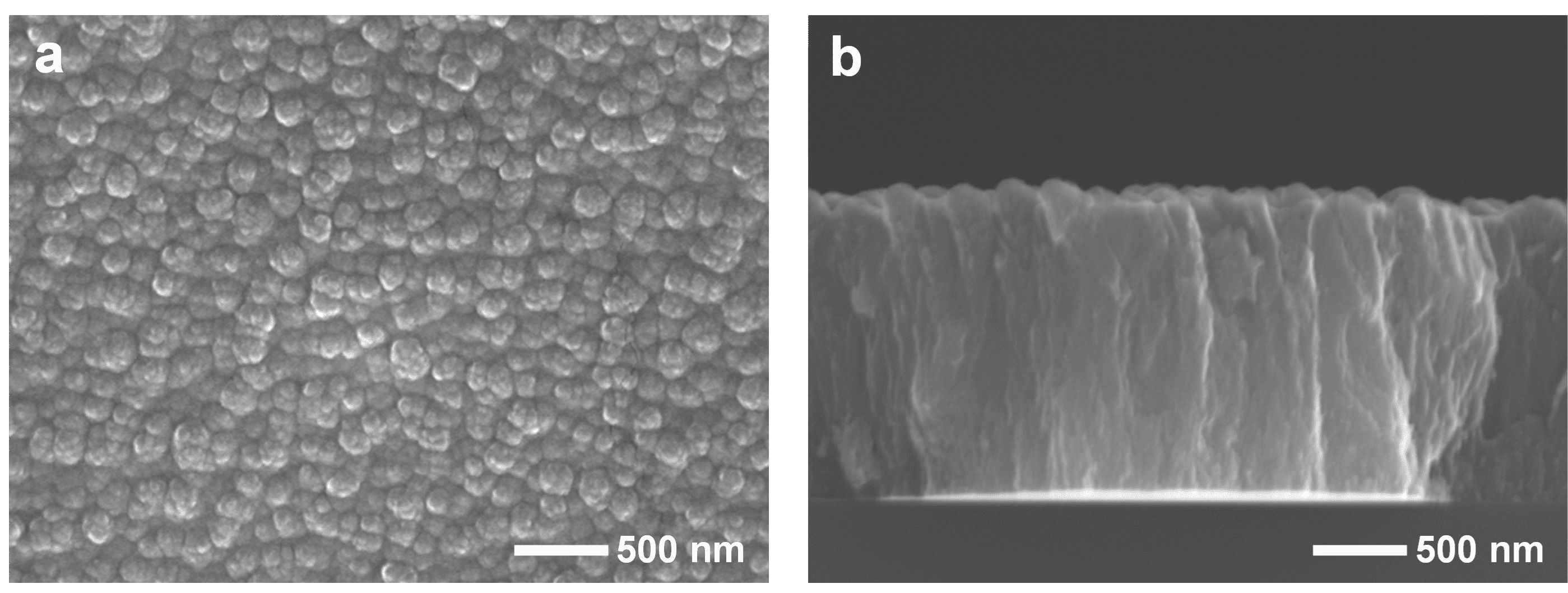


**Figure S7.** a) Top-view and b) cross-sectional SEM images of TiO_2_ film.


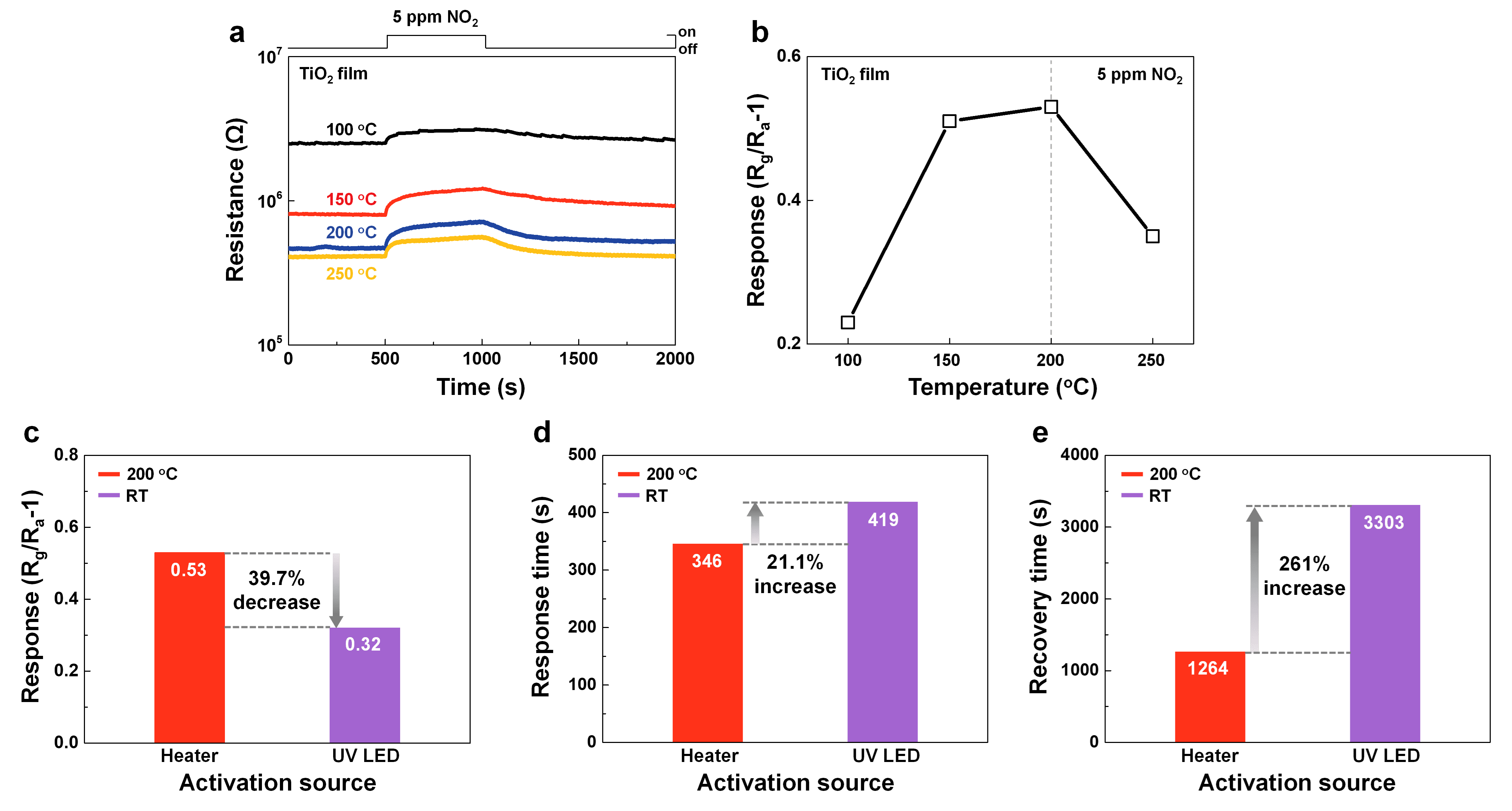


**Figure S8.** a) Resistance curves and b) responses of the TiO_2_ film to 5 ppm NO_2_ at operating temperatures of 100, 150, 200, and 250 °C. Comparisons of c) responses, d) response times, and e) recovery times of the TiO_2_ film under the different activation sources (heater vs. UV LED).


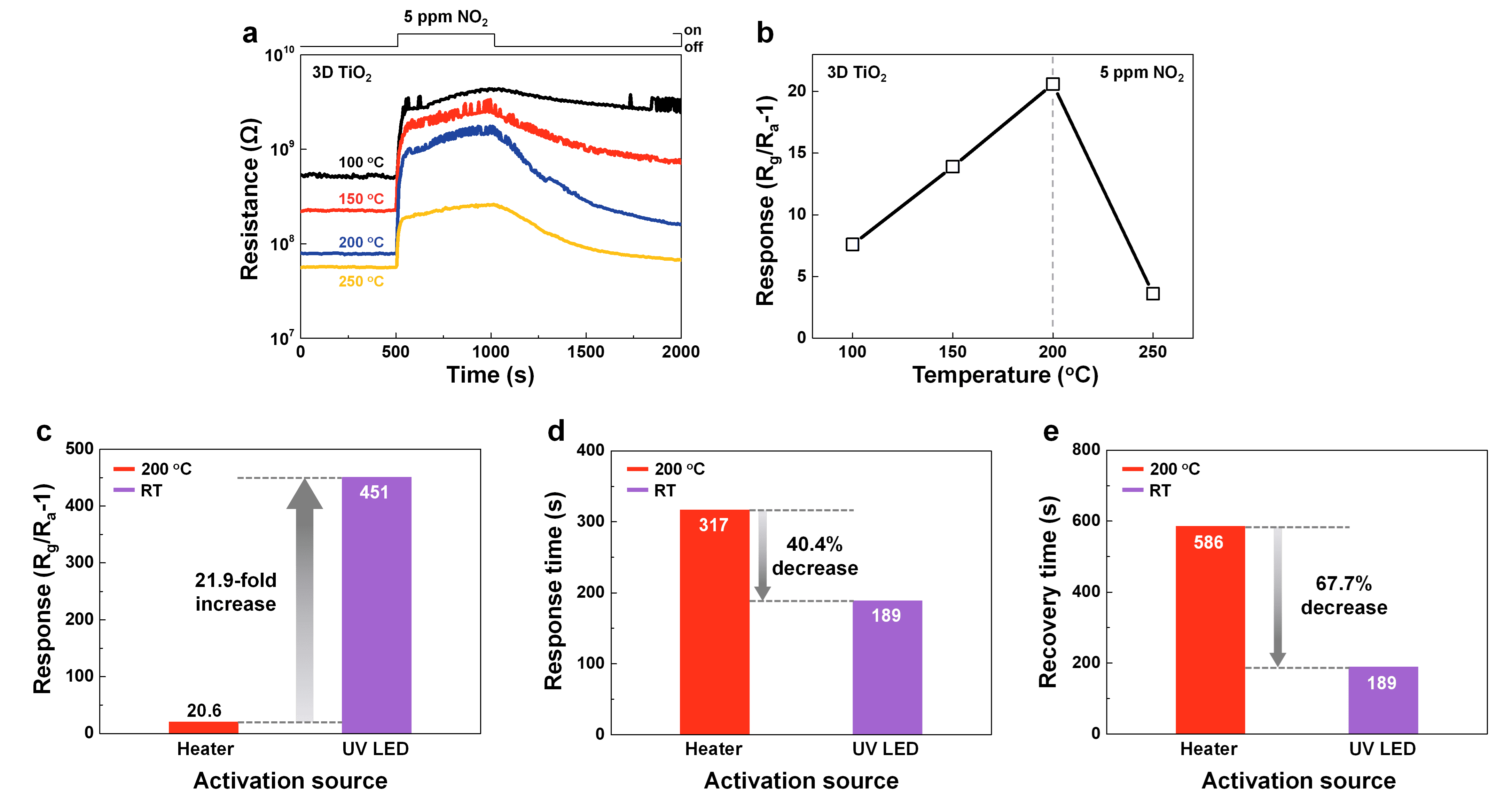


**Figure S9.** a) Resistance curves and b) responses of the 3D TiO_2_ exposed to 5 ppm NO_2_ at operating temperatures of 100, 150, 200, and 250 °C. Comparisons of c) responses, d) response times, and e) recovery times of the 3D TiO_2_ under the different activation sources (heater vs. UV LED).


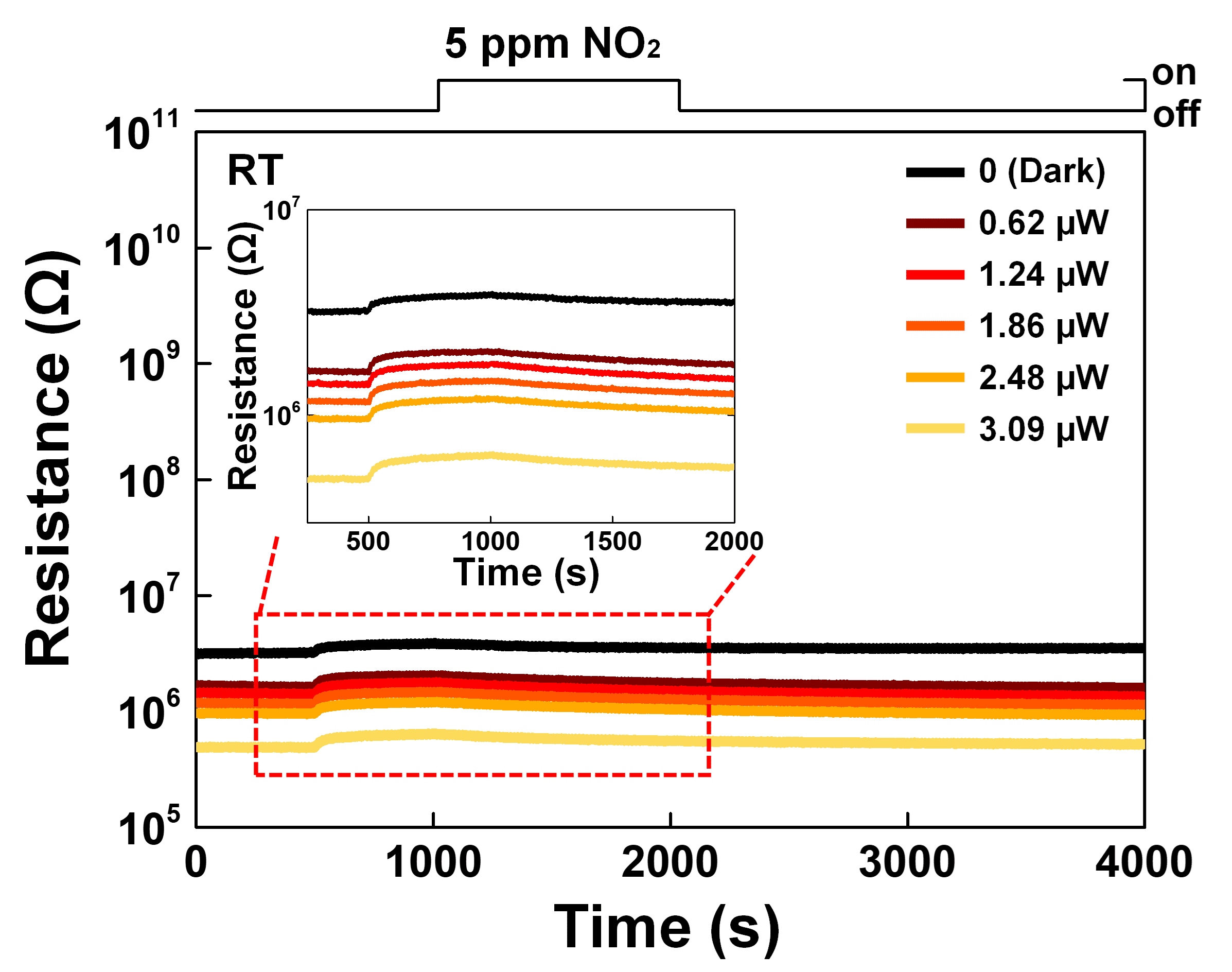


**Figure S10.** Resistance curves of the TiO_2_ film exposed to 5 ppm NO_2_ under UV illumination at light intensities of 0, 0.62, 1.24, 1.86, 2.48, and 3.09 µW.


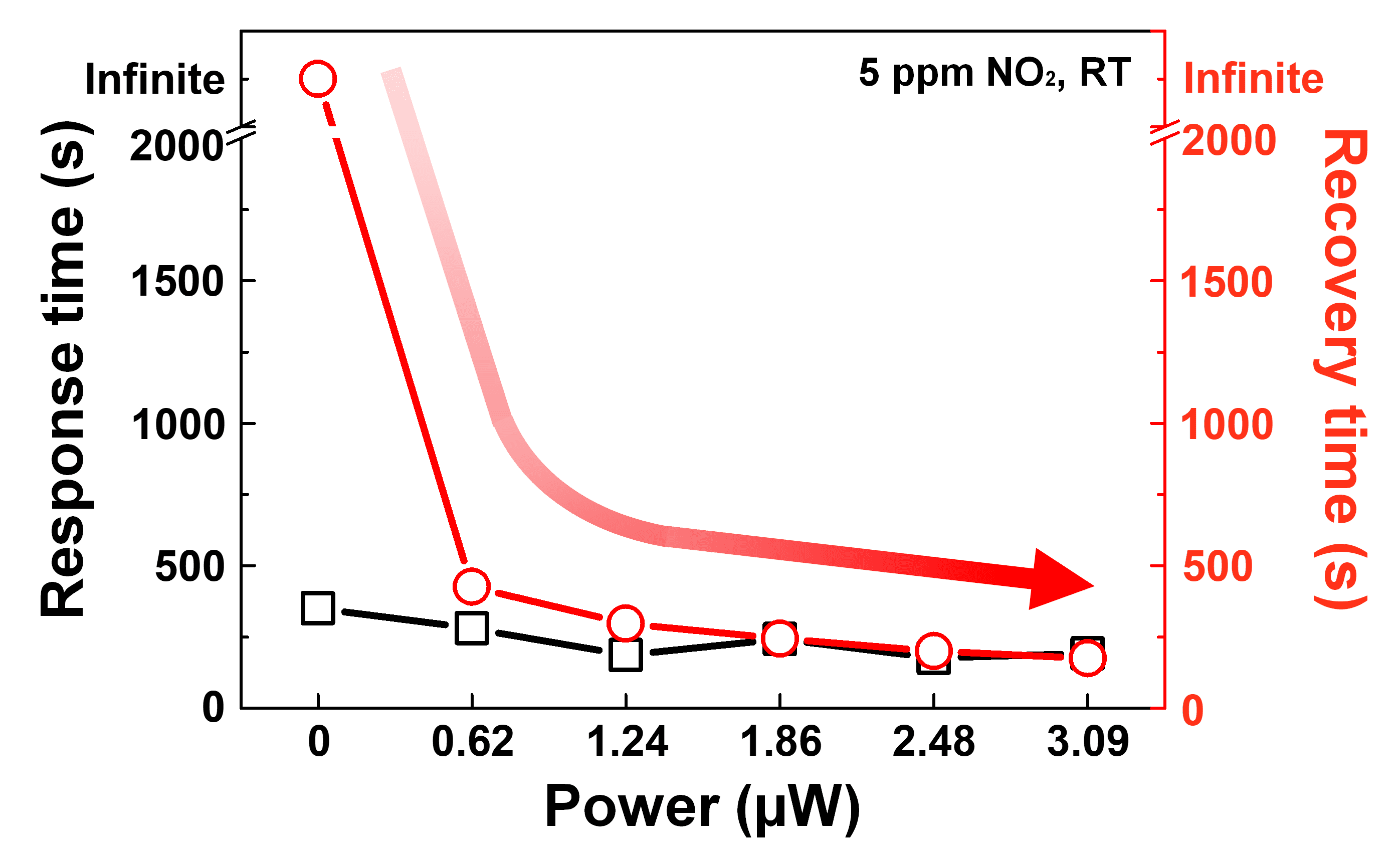


**Figure S11.** Response and recovery times of 3D TiO_2_ exposed to 5 ppm NO_2_ under UV illumination at light intensities of 0, 0.62, 1.24, 1.86, 2.48, and 3.09 µW.


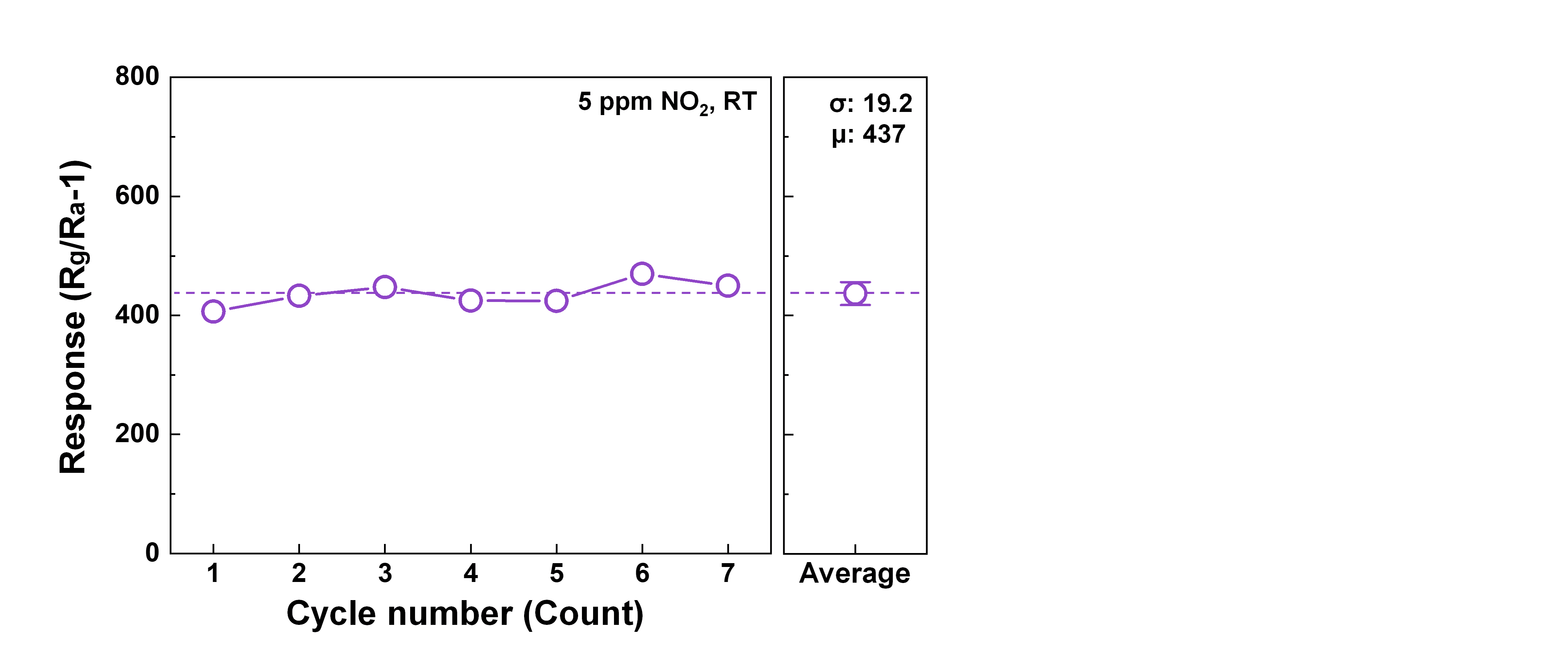


**Figure S12.** Summary of seven cyclic responses and the corresponding average response of the 3D TiO_2_ to 5 ppm NO_2_ under UV illumination.


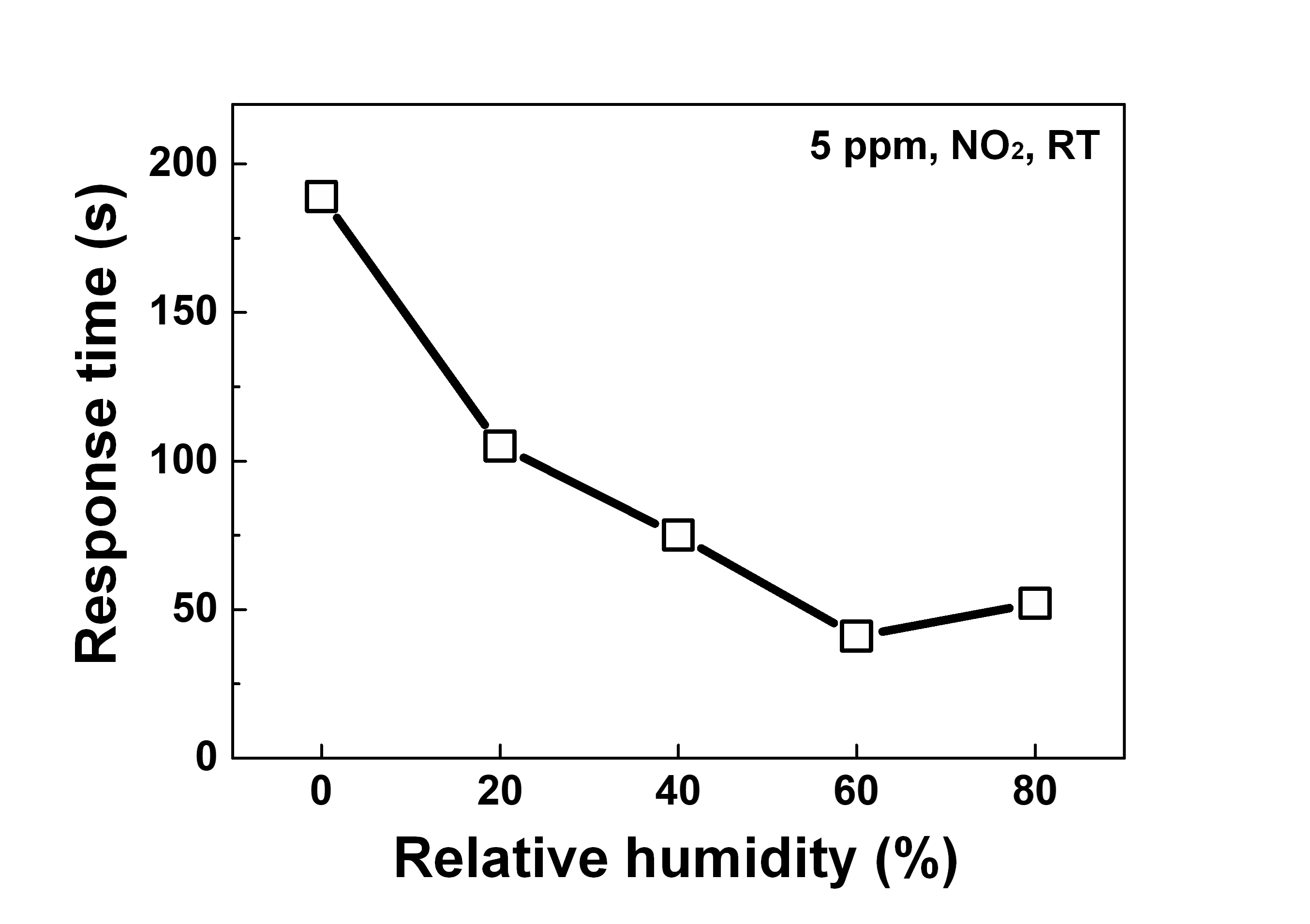


**Figure S13.** Response times of 3D TiO_2_ exposed to 5 ppm NO_2_ under UV illumination at RH levels of 0, 20, 40, 60, and 80%.


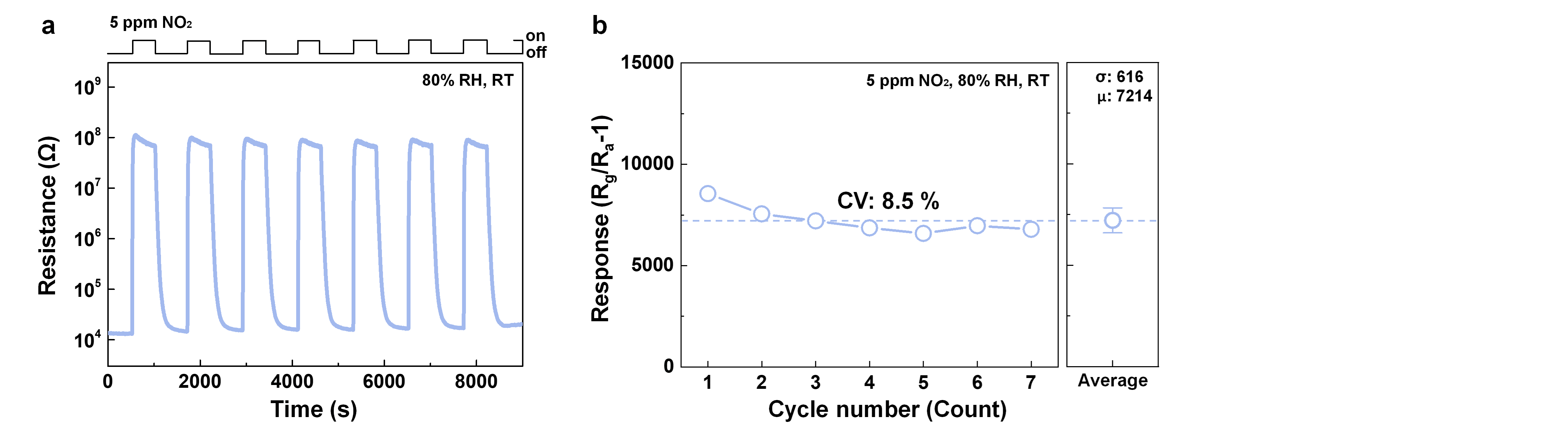


**Figure S14.** a) Cyclic resistance curves, (b) responses and the corresponding average response of the 3D TiO_2_ to 5 ppm NO_2_ under UV illumination in 80% RH condition.


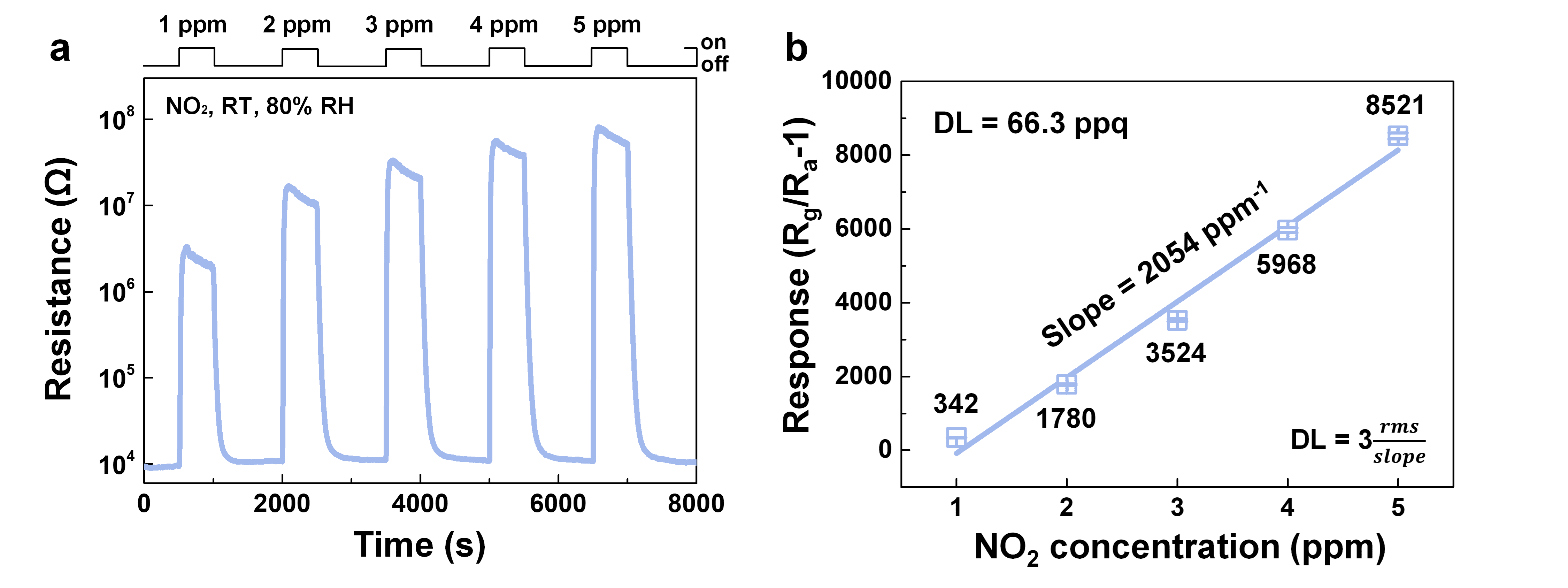


**Figure S15.** a) Resistance curves and b) linear fit of the responses of 3D TiO_2_ exposed to 1–5 ppm NO_2_ under UV illumination at 80% RH condition.


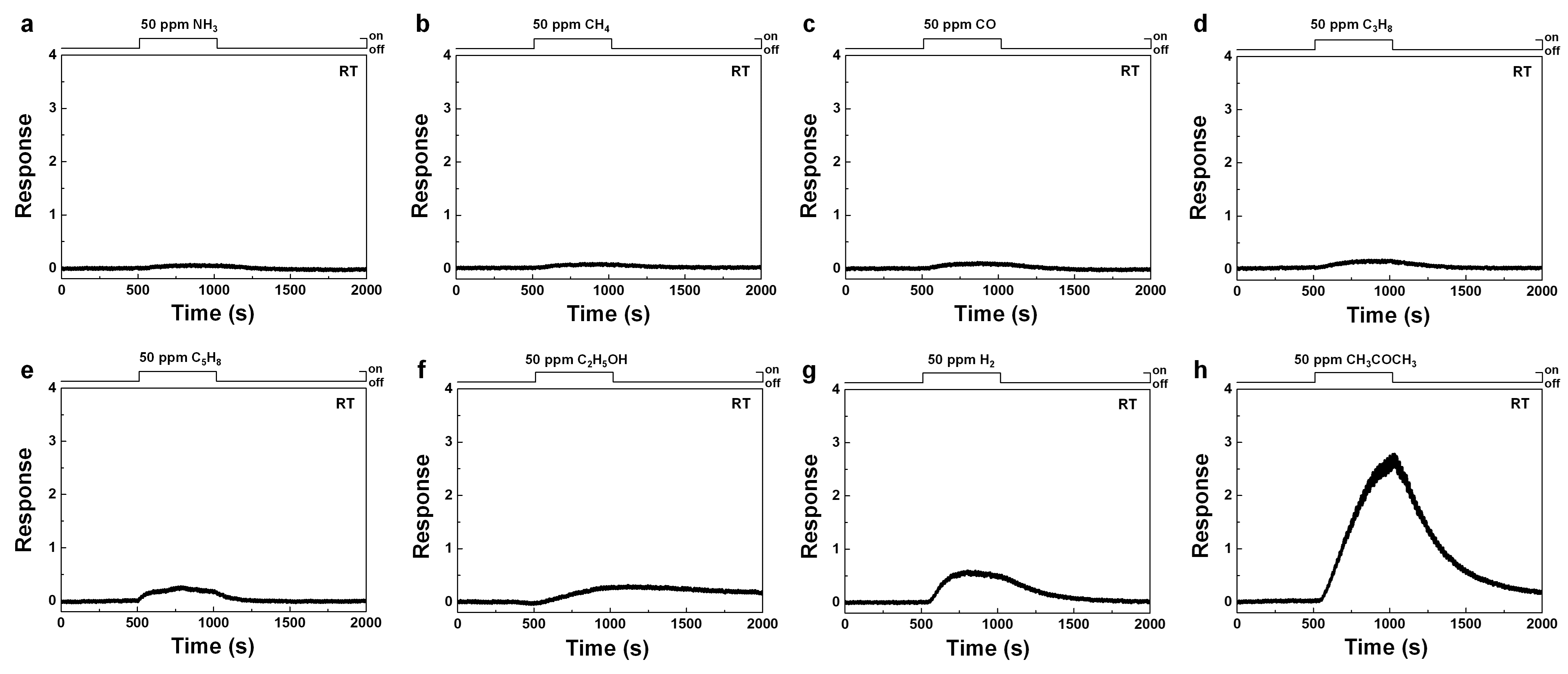


**Figure S16.** Response curves of 3D TiO_2_ to 50 ppm of a) NH_3_, b) CH_4_, c) CO, d) C_3_H_8_, e) C_5_H_8_, f) C_2_H_5_OH, g) H_2_, and h) CH_3_COCH_3_ under UV illumination.


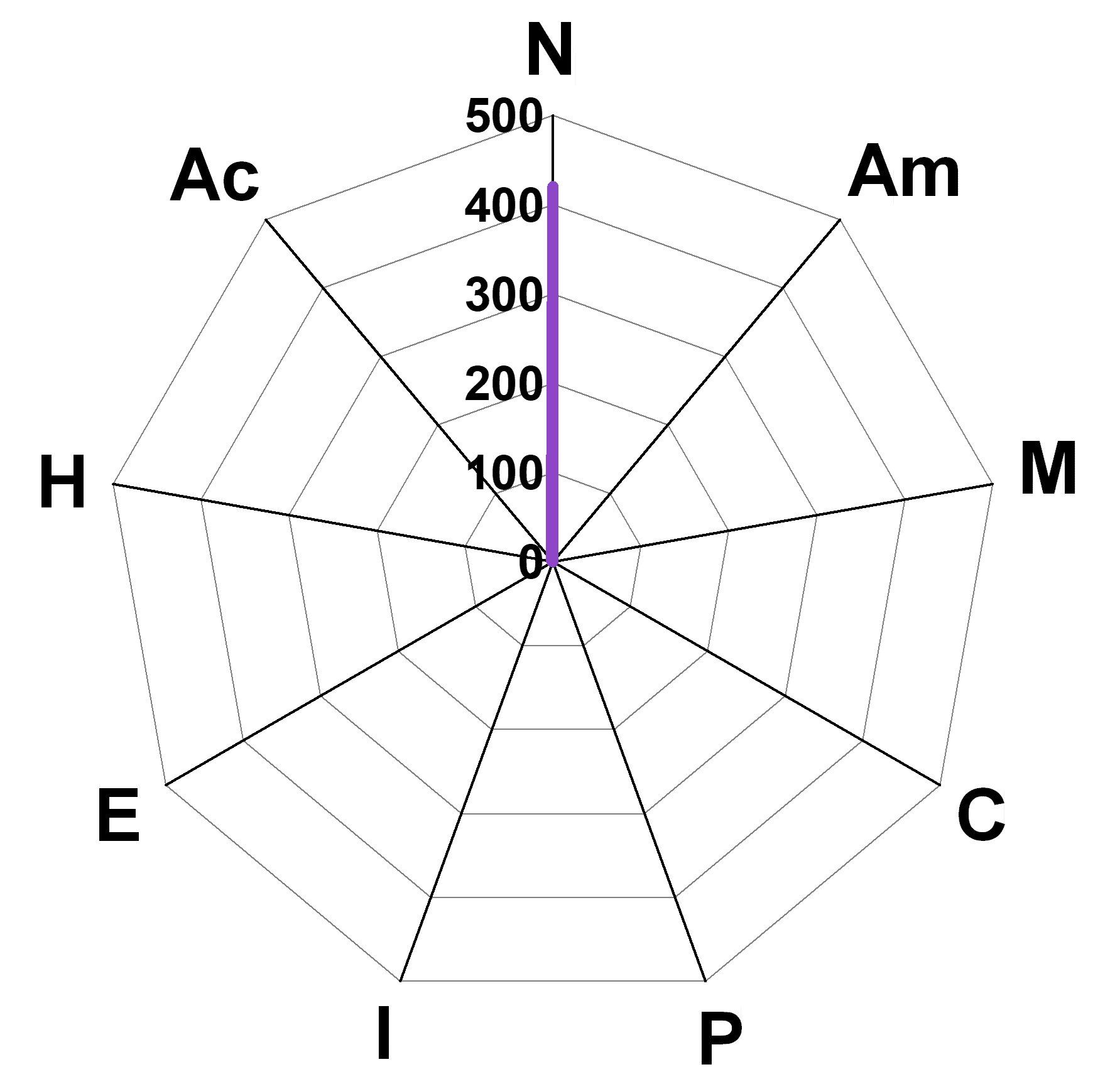


**Figure S17.** Response radar plots of 3D TiO_2_ exposed to 5 ppm NO_2_ and 50 ppm of eight interfering gases (N: nitrogen dioxide, Am: ammonia, M: methane, C: carbon monoxide, P: propane, I: isoprene, E: ethanol, H: hydrogen, and Ac: acetone).


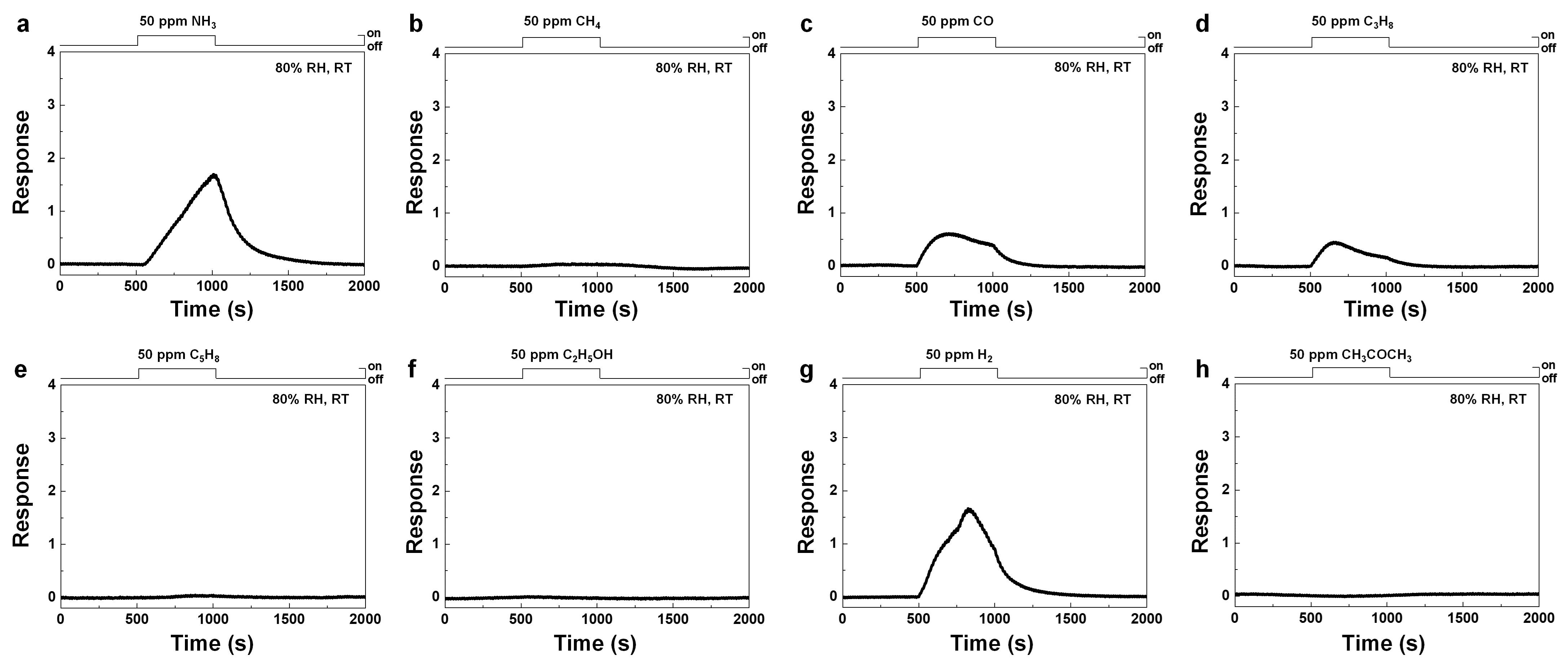


**Figure S18.** Response curves of 3D TiO_2_ exposed to 50 ppm of a) NH_3_, b) CH_4_, c) CO, d) C_3_H_8_, e) C_5_H_8_, f) C_2_H_5_OH, g) H_2_, and h) CH_3_COCH_3_ under UV illumination at 80% RH.


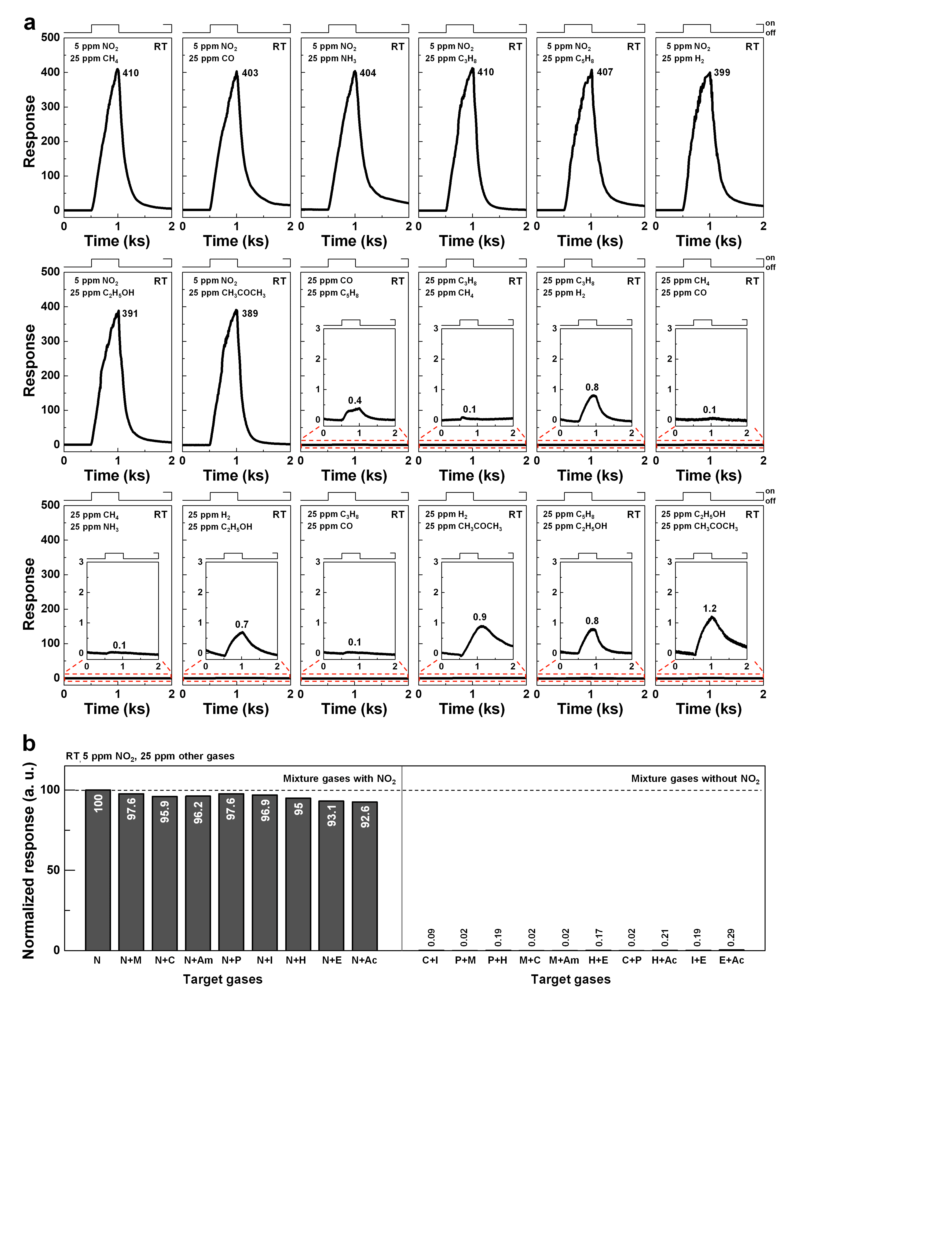


**Figure S19.** a) Response curves of 3D TiO_2_ to different mixture gases: eight mixtures containing 5 ppm NO_2_ and 25 ppm of interfering gases, and ten mixtures containing 25 ppm of two interfering gases (N: nitrogen dioxide, Am: ammonia, M: methane, C: carbon monoxide, P: propane, I: isoprene, E: ethanol, H: hydrogen, and Ac: acetone). b) Normalized responses for eighteen types of mixture gases.


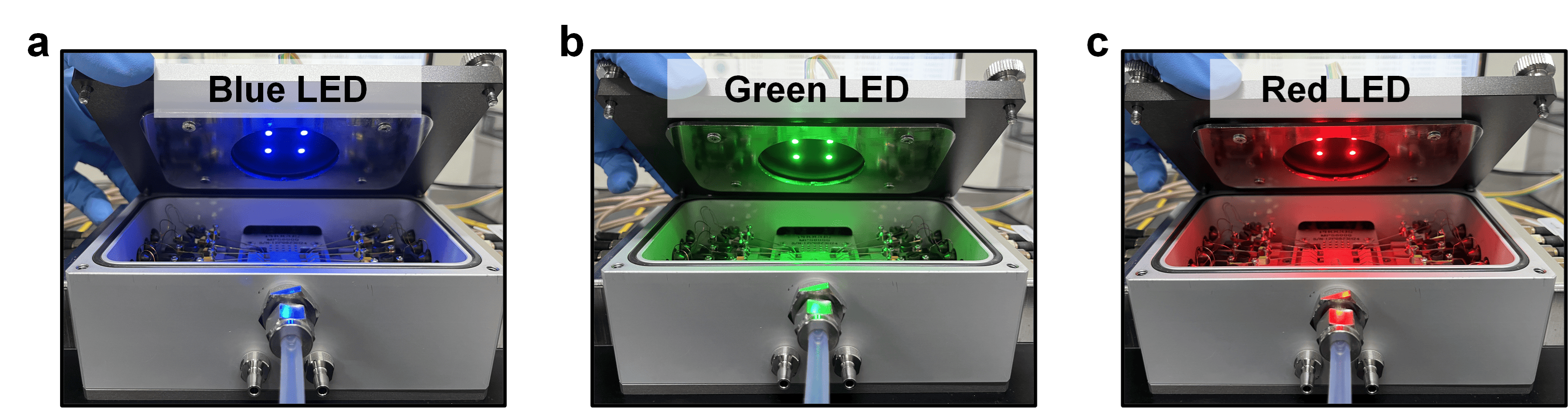


**Figure S20.** Photographs of the experimental setup for photoactivated chemiresistive gas sensing under a) blue, b) green, and c) red LED illumination.

**Figure S21.** Summary of responses and response/recovery times of 3D TiO_2_ exposed to 5 ppm NO_2_ under RGB illumination in a–c) dry and d–f) 80% humid conditions.


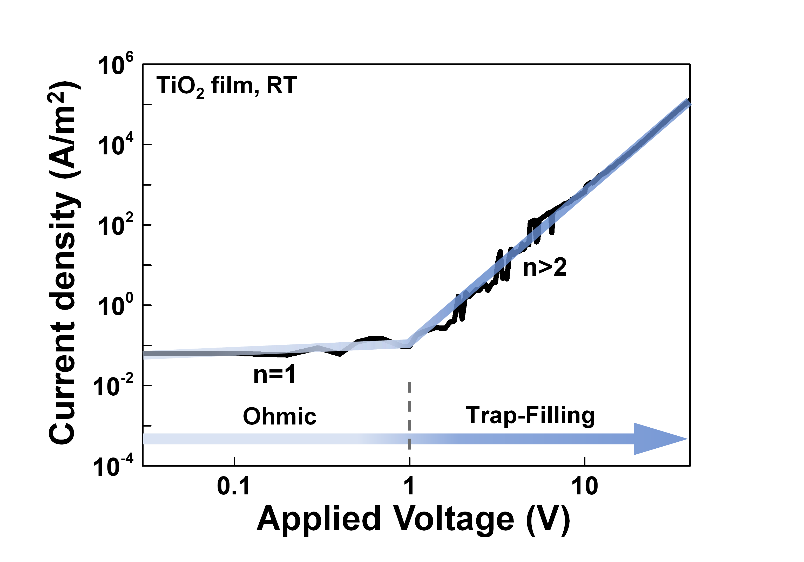


**Figure S22.** Log J vs. log V characteristics of the TiO_2_ film.


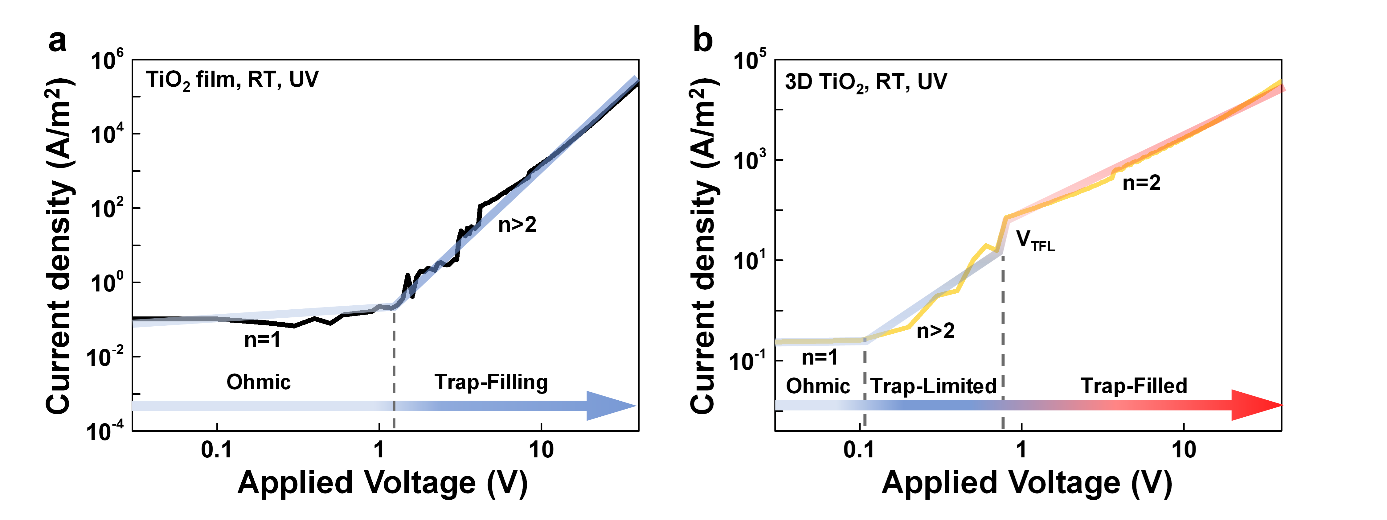


**Figure S23.** Log J vs. log V characteristics of a) the TiO_2_ film and b) 3D TiO_2_ under UV illumination.


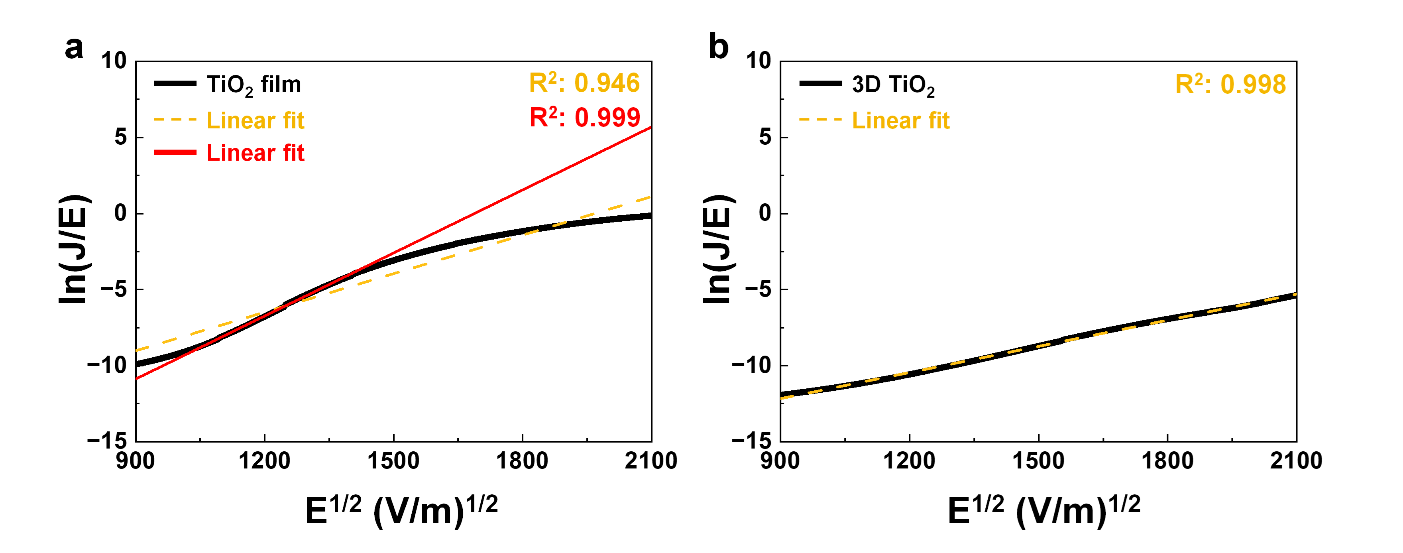


**Figure S24.** ln(J/E) vs. E^1/2^ characteristics of a) the TiO_2_ film and b) 3D TiO_2_.


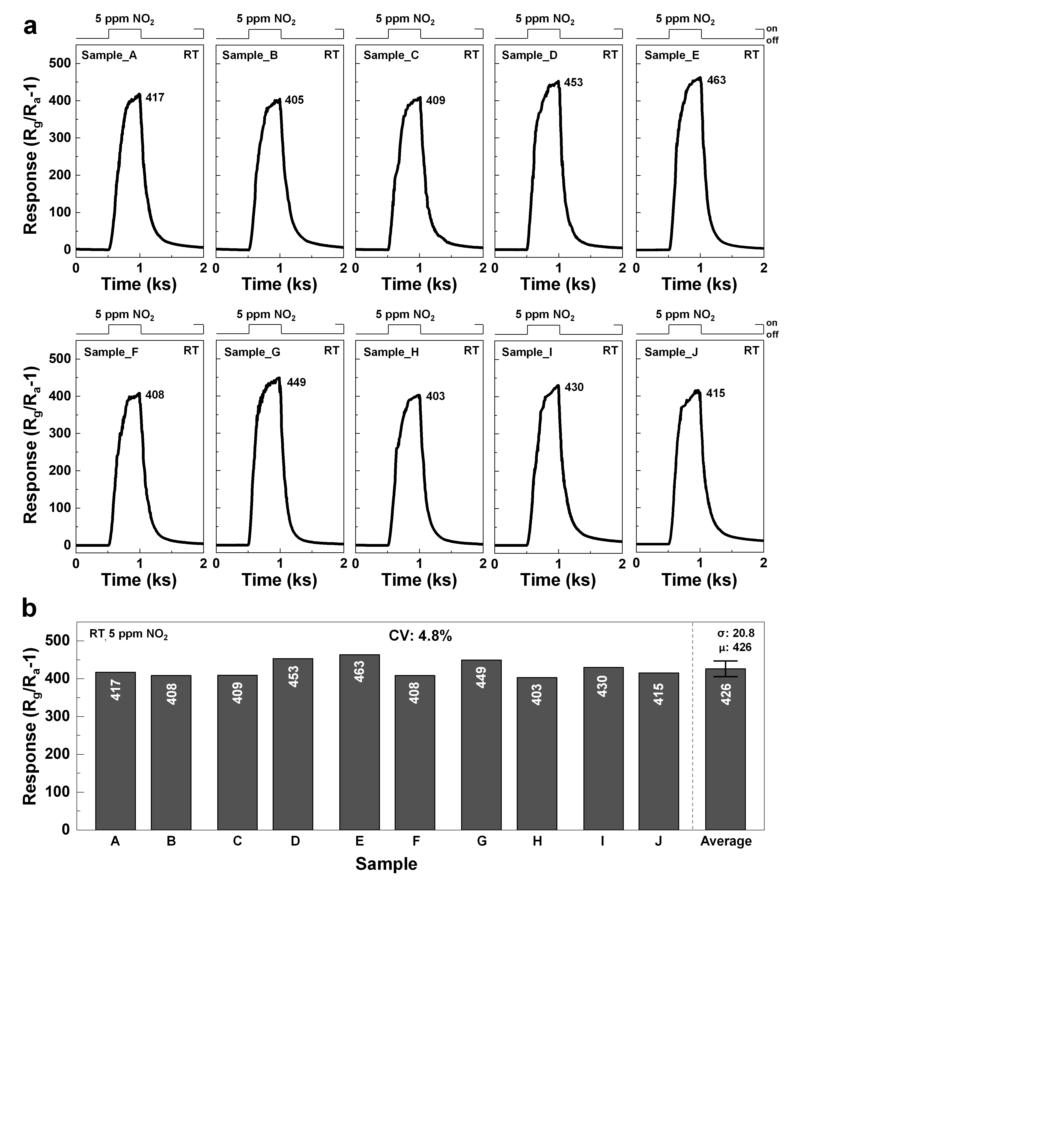


**Figure S25.** a) Response curves and b) corresponding responses to 5 ppm NO_2_ for ten sensors that were fabricated on a 4-inch wafer substrate.


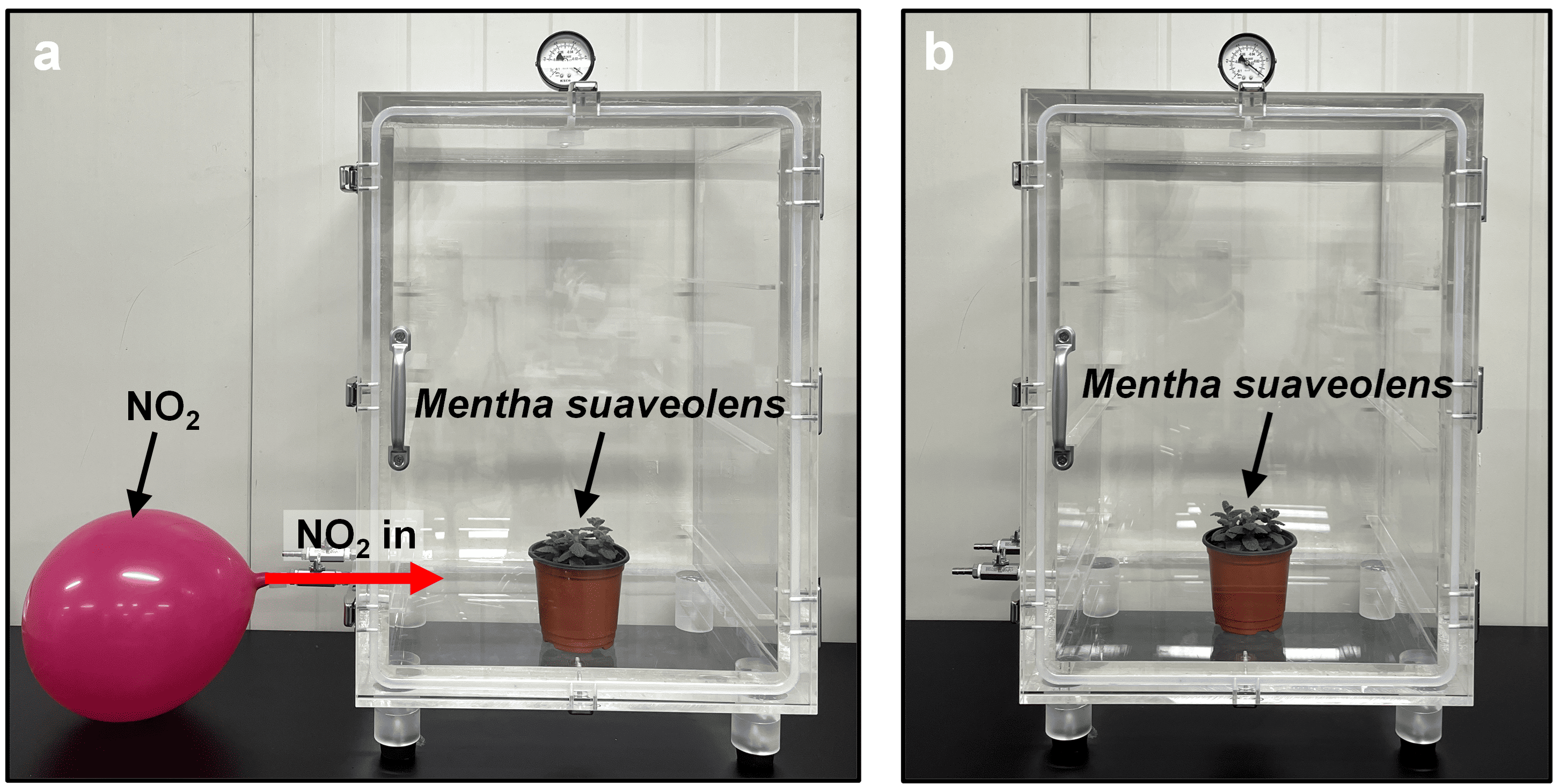


**Figure S26.** a,b) Photographs of the experimental setup to assess phytotoxic effects of 20 ppm NO_2_ exposure.


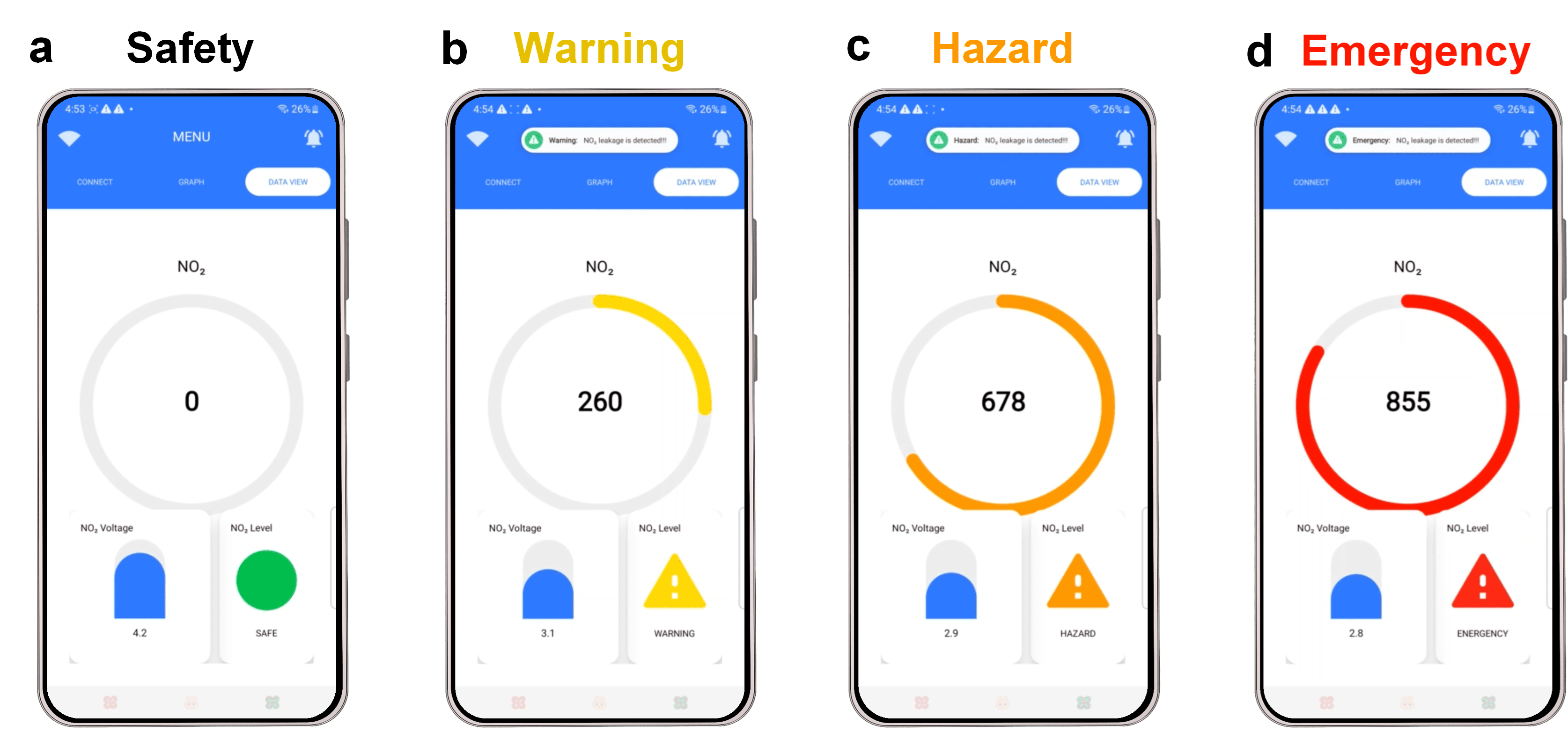


**Figure S27.** a–d) User interfaces of a gas notification application with different risk levels based on NO_2_ concentrations.

**Figure S28.** Resistance curves of 3D TiO_2_ to NO_2_ under a) sunlight and b) indoor illumination, measured using a real-time NO_2_ sensing platform.


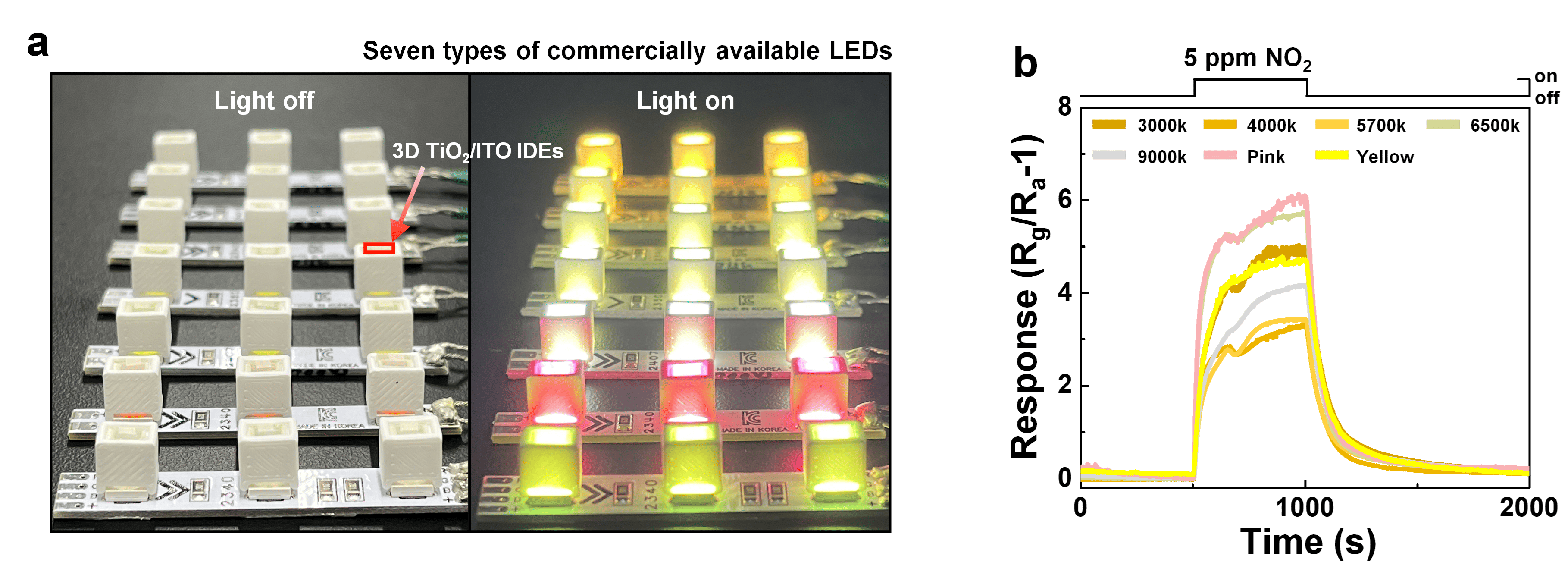


**Figure S29.** a) Photographs of 3D TiO_2_/ITO IDEs on seven types of commercial LEDs. b) Response curves of 3D TiO_2_ exposed to 5 ppm NO_2_ under each LED illumination.

**Table S1.** Comparative summary of light-activated NO_2_ sensing performances toward 5 ppm NO_2_.

| **Sensing materials** | **Light source** | **RH**  **(%)** | **Response** | **DL**  **(ppt)** | **Response time (s)** | **Recovery time (s)** | **Ref.** |
| --- | --- | --- | --- | --- | --- | --- | --- |
| 3D thin-shell TiO_2_ | UV | Dry | 31 | 202 | 428 | – | [30] |
|  |  | 50 | 115 | – | – | – |  |
|  | Blue | Dry | 4 | – | – | – |  |
|  | Green | Dry | 1 | – | – | – |  |
|  | Red | Dry | 3 | – | – | – |  |
| SnO_2_ NPs | Blue | Dry | 158 | – | – | – | [47] |
| S-SnO_2_ NPs | Green | Dry | 59 | 13 | 405 | 555 |  |
|  | Red | Dry | 11 | 2360 | 461 | 1919 |  |
| Ag NP coated-In_2_O_3_ TF | Blue | Dry | 60 | 2900 | 1320 | – | [51] |
| SnO_2_ NF-mats | Blue | Dry | 2 | – | – | – | [52] |
|  | Green | Dry | 2 | – | – | – |  |
|  | Red | Dry | 2 | – | – | – |  |
| Au-SnO_2_ | Blue | Dry | 180 | 6000 | 500 | 223 |  |
| WO_3_ nano-particles | UV | Dry | 17 | – | – | – | [53] |
| ZnO/In_2_O_3_ composite | UV | Dry | 2 | – | – | – | [54] |
| 3D TiO_2_ | UV | Dry | 420 | 45 | 189 | 222 | This  work |
|  |  | 80 | 8521 | 0.066 | 62 | 33 |  |
|  | Blue | Dry | 312 | 72 | 441 | 694 |  |
|  |  | 80 | 2102 | 11 | 125 | 45 |  |
|  | Green | Dry | 70 | 500 | 424 | 1958 |  |
|  |  | 80 | 9748 | 6 | 178 | 46 |  |
|  | Red | Dry | 45 | 1800 | 437 | > 5000 |  |
|  |  | 80 | 28322 | 3 | 157 | 42 |  |
